# Enhanced biosensing of diabetes biomarkers by using rationally engineered carbon dots

**DOI:** 10.1039/d5ra08089d

**Published:** 2026-03-23

**Authors:** Lina Anil Kumar, Kalyani Priya Manoj, Ananya Kannamvelli Illam, Angel Mariyam Biju, Vaishnavi Mekkeparambath, M. M. Sreejaya, Teresa Aditya, Moumita Gangopadhyay

**Affiliations:** a Amrita Vishwa Vidyapeetham Amritapuri Kollam 690525 India moumita@am.amrita.edu; b Pennsylvania State University State College PA 16801 USA teresaa@psu.edu

## Abstract

Carbon dots are quasi-spherical zero-dimensional nanomaterials. Small size and tunable optical properties render carbon dots with excellent biosensing ability. They exhibit fascinating excitation-wavelength-dependent emission properties, attributed to their sp^2^-carbon core. Presence of dopants alters the energy levels of carbon dots, improving their optical properties and their facile surface functionalization with suitable electron donor/acceptor groups or biomolecules results in modification of their electronic properties, which in turn facilitates fluorescence-based detection of significant biomarkers. Biocompatibility and hydrophilicity, along with their captivating optical properties, inspired researchers worldwide to utilize carbon dots in the timely detection of diabetes biomarkers. Diabetes has long been one of the more concerning diseases. It shows long-term adverse effects, harming vital organs like kidney, heart, eyes, *etc.* Therefore, it must be diagnosed at its inception stage. Impaired metabolism of insulin in diabetic patients elevate concentrations of several biomolecules like glucose, reactive oxygen species, *etc.*, which can be detected by fluorescence. The widespread application of fluoresce sensing in biomedicine stems from its non-invasive nature and precision. This review article highlights the influence of doping and surface functionalization of carbon dots on their improved selectivity and biocompatibility toward biosensing of diabetes markers.

## Abstract 2

Appropriate surface-decorated carbon dots (CDs) for fluorescence sensing of diabetes biomarkers like elevated levels of glucose and reactive oxygen species.

## Graphical abstract

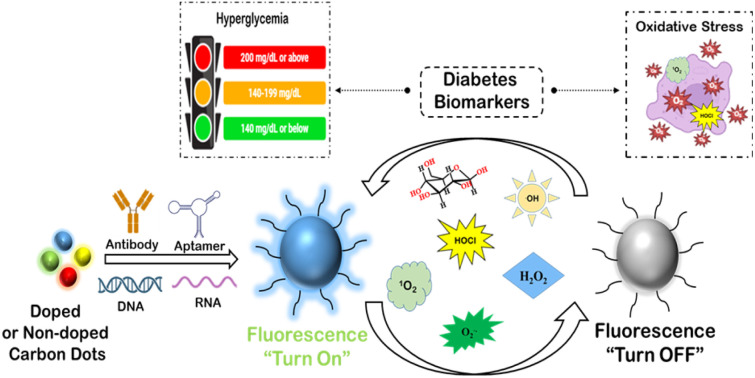

## Introduction

Carbon dots are zero-dimensional nanoparticles with sizes in the range of 0–10 nm and show intriguing optical properties.^1^ Their excellent absorption property extends up to the visible region and sometimes the near-infrared (NIR) region,^2–4^ which is attributed to their sp^2^-carbon-rich core structure with extensive conjugation. Carbon dot cores often display π–π* and *n*–π* transitions owing to the sp^2^-hybridized π-conjugated structure and diverse surface functionalities, respectively. The extended absorption range of carbon dots leads to their excitation-wavelength-dependent emission properties, which is directed by their inherent energy gap and nitrogen or oxygen defect sites based on the carbon source from which they are synthesized.^5,6^ To improve their optical properties, carbon dots are frequently doped with various substances like nitrogen, sulfur, different metals, *etc.*^7–9^ Doping carbon dots with non-metallic heteroatoms like nitrogen, sulfur, boron, phosphorous, *etc.* led to the formation of more defect sites in the carbon dot structure, which in turn alters the distribution of electrons in the carbon dots. Moreover, the closer the size of the heteroatom is to that of the carbon atom, better is the overlap between the atomic orbitals of carbon and the heteroatom, thereby amplifying the optical responses.^7^ Metal atoms having greater electron density, larger size, vacant orbitals, and better polarizability undergo better conjugation with carbon atoms, leading to reduction in the energy gap for carbon dots.^8^ Besides singly doped carbon dots, co-doping has shown promising results in improving the quantum yield of carbon dots.^10,11^ Exciting optical properties, such as longer absorption wavelength, greater emission intensity, increased quantum yield, *etc.* make the doped carbon dots suitable for application in fluorescence-based detection of biomarkers.^1–3^ Additionally, facile functionalization of carbon dots with biomolecules like antibodies, aptamers, *etc.* enhances its cellular penetration, bioavailability, hydrophilicity, and target specificity, making carbon dots suitable for application in the field of biomedicine.^12–15^

Among non-lethal diseases, diabetes has long been a matter of serious concern due to its long-term adverse effects. Diabetes type I and II are usually caused by insulin resistance or impaired production of insulin,^16^ and cause severe damage to kidney, heart, eye, bones, *etc.* Therefore, early detection of diabetes helps control the progression of the disease. Fluorescence-based detection of any disease has attracted more attention than other detection methods, owing to the non-invasiveness, accuracy, and spatio-temporal specificity of fluorescence. There have been several attempts to develop fluorescence sensors to detect prominent biomarkers associated with diabetes, *e.g.*, elevated glucose level, abundant reactive oxygen species, higher methylglyoxal concentration, greater viscosity, *etc.*^17^ Diverse fluorescent probes, based on small organic molecules, polymers, supramolecular structures, nanomaterials, *etc.*, have been exploited for the selective detection of diabetes biomarkers.^18–22^ Carbon dots, due to their high water solubility, non-cytotoxicity, ease of functionalization, and fascinating optical properties have shown great prospect for application in the fluorescence sensing of diabetes-related biomarkers. They can participate in various energy/electron transfer processes with the substituents on its surface, leading to distinct changes in their absorption or emission properties, which indicate the interaction with specific biomarkers. There have been many review articles highlighting the synthesis and origin of emission in carbon dots.^1,23–25^ Further, some of the literature reviews have discussed the application of carbon dots in biosensing, bioimaging, *etc.*^26,27^ However, there has hardly been any review study on the application of carbon dots, specifically in the detection of diabetes-related biomarkers.^28^ Considering the seriousness of the disease, a holistic account of the use of carbon dots in the diagnosis of diabetes is highly relevant.

**Scheme 1:**
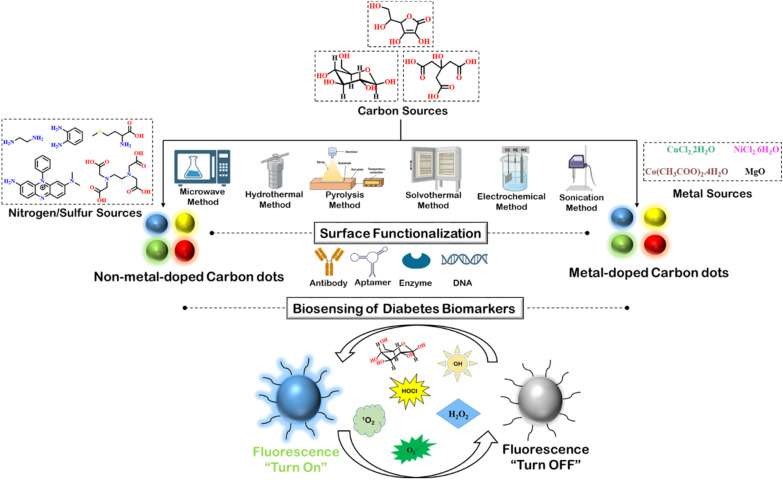
Depiction of versatile carbon dots and its application in detection of diabetes biomarkers.

In this review article, different types of doped, non-doped, and co-doped carbon dots have been exemplified, focusing on their optical properties based on their synthetic methods as shown in Scheme 1. Regulating the optical properties of carbon dots is one of the primary requisites for successful application in the diagnosis of diabetes. Modification of surface functionalities plays an important role in this regard, which has been thoroughly discussed in this report. The significance of conjugating carbon dots with biomolecules like antibody, DNA, aptamer, *etc.*, in terms of bioavailability and specificity, has also been highlighted. Finally, some of the recent studies on fluorescence-based detection of diabetes-associated biomarkers have been discussed with vivid mechanistic details. Therefore, this review article can serve as a timely account of the detection of diabetes by carbon dots.

## Structural and chemical variants of carbon dots (CDs)

The small size of CDs, along with their chemical and structural diversity, make their classification quite challenging. CDs cannot be defined exclusively in terms of its chemical, structural, or functional properties. The prevailing consensus is to categorize CDs based on their composition and structure. Bare or pristine CDs are classified as carbon quantum dots (CQDs), graphene quantum dots (GQDs), and polymer carbon dots (PCDs) based on the source of the carbon.^29–32^ However, owing to their distinctive structural features, like the layered graphene structures, sp^2^-hybridized carbon atoms, larger lateral dimension, *etc.*, GQDs usually stand alone. On the other hand, the CQDs contain crystalline lattice and spherical structures, while PCDs show amorphous lattice structure. Besides these, functionalized or doped CDs have attracted considerable research attention due to their exceptional optical, catalytic, and electronic properties.^8,33–37^ In this section, properties and effective synthetic protocols of several types of CDs have been discussed.

### Pristine carbon dots (CDs)

CDs are usually synthesized following both top-down and bottom-up approaches. In the top-down approach, bulkier carbon-based materials like graphite, carbon-containing fibers, graphene oxide, *etc.* are broken into smaller dots following methods like electrochemical, acidic digestion, *etc.*^38,39^ The superiority of this top-down approach lies in its cost-effectiveness, shorter reaction time, and easy scaling-up nature. However, the bottom-up approach involves lengthier and critical reactions between small carbon precursors like citric acid, glucose, or other aromatic hydrocarbons. The choice of synthetic method depends on the required shape, size, and property of the CDs.

**Fig. 1:**
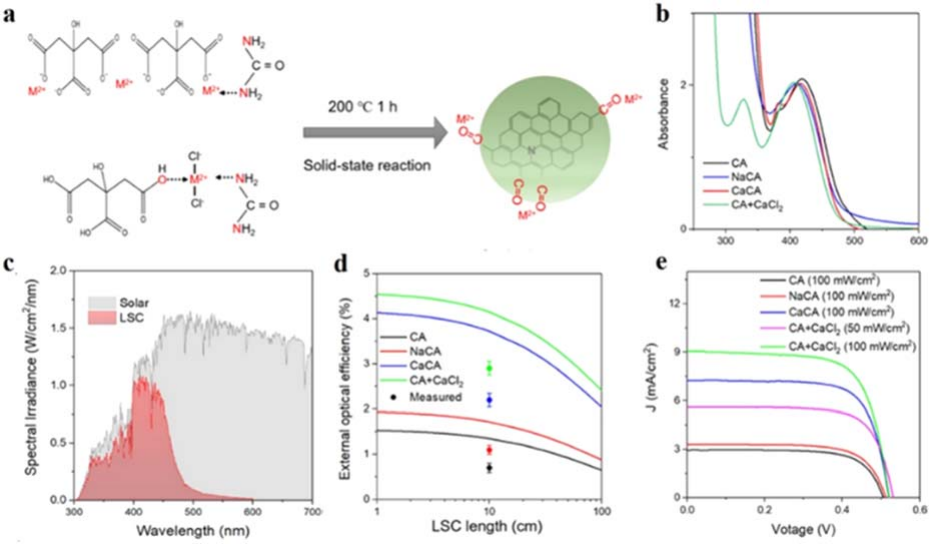
(a) Synthesis of carbon dots *via* solid state reaction. (b) Absorbance, (c) comparative spectral irradiance of LSC, (d) external optical efficiency of LSC, and (e) comparative voltage output of different CDs. [Reprinted from Bingxu Liu, Lihua Wang, Xiao Gong, Haiguang Zhao, Yuanming Zhang, Large scale synthesis of carbon dots for efficient luminescent solar concentrators, *J. Mater. Chem. C*, **10**, 18154–18163, copyright (2022), with permission from Royal Society of Chemistry].^40^

In a recent finding, CDs synthesized following a bottom-up approach by heating carbon precursors under solvent-free condition, have been used for developing luminescent solar collectors (LSC) (Fig. 1).^40^ In their study, Liu *et al.* mixed citric acid and urea in a mortar pestle at room temperature. After grinding and mixing the precursors in a 1 : 2 weight ratio for ∼10 min, the mixture was subjected to oven-heating at 200 °C for 1 h. Liu *et al.* also synthesized metal-capped CDs in larger scale for better solar energy conversion. For synthesizing different metal-capped CDs, a series of metal chlorides were added to 1 : 2 mixtures of citric acid and urea, maintaining 1 : 1 weight ratio with respect to citric acid. Owing to the high surface passivation, these CDs showed excellent quantum yields, making them suitable for application in LSCs.

Apart from their extraordinary optical properties, several un-doped CDs have shown important biological effects like mimicking enzymatic activities.^41^ In a recent study by Lin *et al.*, novel citric-acid-derived CDs have been synthesized, which can mimic cysteine oxidase, an enzyme that controls the functioning of the essential amino acid, cysteine. To achieve the best biocatalytic activity, the CDs were synthesized from naturally occurring citric acid following a simple solvothermal pyrolysis method. The CDs, obtained upon heating at 200 °C, showed excellent water solubility and significant binding affinity towards cysteine with a fluorescence turn-on. The detection limit was found to be as low as 0.036 µM.

**Fig. 2:**
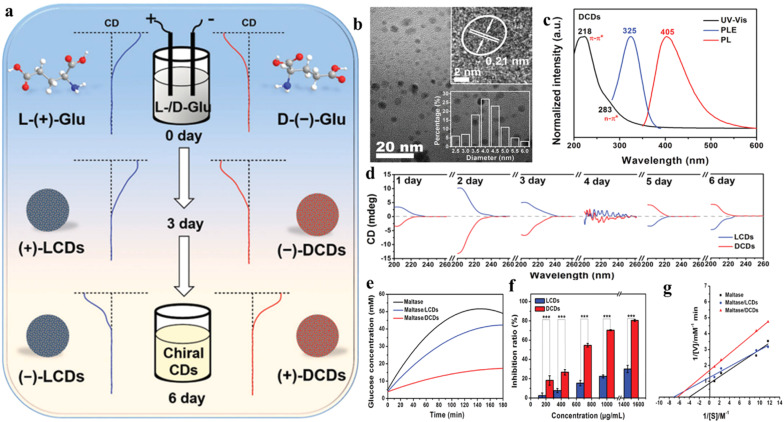
(a) Synthetic route for chiral carbon dots, characterization of carbon dots using (b) TEM imaging and (c) absorption–emission spectral analysis, (d) circular dichroism (CD) spectra of carbon dots prepared with different electrolysis times, (e) monitoring change in the concentration of glucose to elucidate maltase inhibition, (f) comparative maltase inhibition ratio, and (g) Lineweaver–Burk plot. [Reprinted from Zhenhui Kang, Bowen Yao, Mingwang Shao, *et al.*, Maltase decorated by chiral carbon dots with inhibited enzyme activity for glucose level control, *Small*, **15**, 1901512, copyright (2019), with permission from Wiley].^42^

Another recently developed novel class of CDs are chiral CDs. These have found noteworthy application in crucial biological processes like DNA rearrangement, chiral biocatalysis, multi-color bioimaging, *etc.*^25^ The chirality can be incorporated in the CDs by introducing chiral defects, chiral shapes, chiral environment, or ligand-induced energy structure.^25^ For the synthesis of chiral CDs, several top-down and bottom-up approaches have been employed. Electrochemical synthesis of chiral CDs from chiral carbon precursors is one of the more cost-effective and rapid methods. For example, Zhang *et al.* synthesized chiral CDs from the electrochemical exfoliation of l- and d-glutamic acid from a NaOH solution (Fig. 2).^42^ The prepared CDs were smaller than 7 nm. However, CDs obtained by this method contained small amounts of nitrogen-doped CDs as well. Various bottom-up approaches involved employing hydrothermal methods using l-/d-cysteine as precursors.

Apart from the traditional hydrothermal method, other cost-effective and non-hazardous methods like microwave synthesis have been utilized for the synthesis of chiral CDs. In this context, Victoria *et al.* synthesized chiral CDs from a 1 : 1 mixture of citric acid and l-cysteine as the chiral precursor.^43^ The mixture was heated at 220 °C under microwave irradiation for 15 min in the presence of water to produce chiral CDs of the size of 12 nm. The mechanism of forming chiral CDs involved multiple condensation reactions producing 5-oxo-3,5-dihydro-2*H*-thiazolo[3,2-*a*]pyridine-3,7-dicarboxylic acid (TPDCA), which in turn underwent polymerization and carbonization to produce small chiral CDs.

In spite of having fascinating catalytic and optical properties, pristine CDs suffered from low quantum yield, which limits their application. Some of the routes to synthesizing pristine CDs involve critical post-synthetic purification. Additionally, high concentrations of some starting materials often render the pristine CDs with some toxic traits. To address these issues, new types of CDs need to be explored. Doped CDs are emerging as promising alternatives with improved energy gaps, shapes, and sizes, leading to boosted optical, enzymatic, catalytic properties.

### Doped carbon dots

#### Nitrogen-doped carbon dots

Of late, nitrogen-doped carbon dots (NCDs) have garnered significant attention owing to their improved fluorescence quantum yields. Such improvement of photoluminescence behavior is derived from strong doping between carbon and nitrogen owing to their comparable sizes. Higher electronegativity and the available lone pairs on the nitrogen atom enable them to bond well with the carbon sources. Most of the NCDs are preferably prepared following the bottom-up approach, involving reaction between small molecular carbon precursors and nitrogen sources *via* simple convenient methods like microwave-assisted synthesis, carbonization, nitrogenization, hydrothermal or acidic oxidation methods, leading to better yields protocols. On the other hand, the top-down approach often includes arc discharge, electrochemical oxidation reaction, or laser ablation of larger carbon precursors into smaller dot size.

**Fig. 3:**
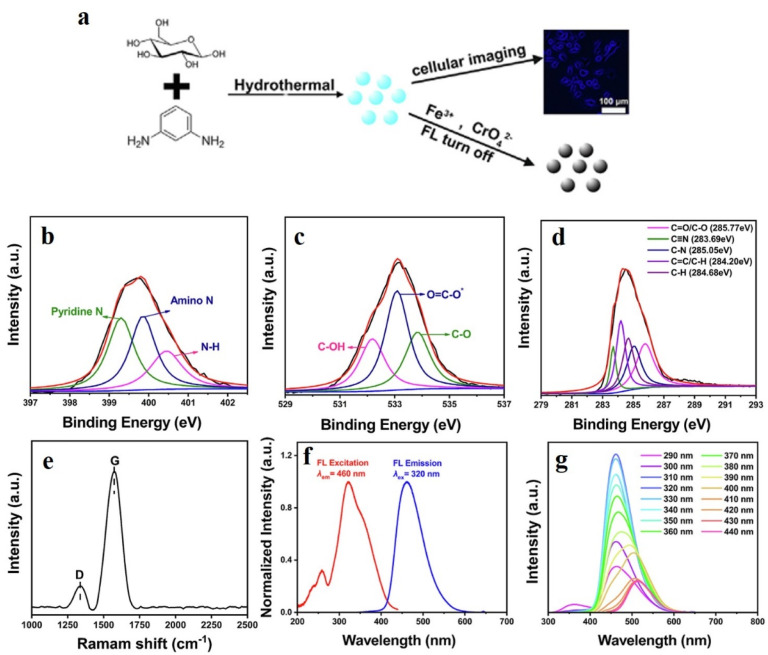
(a) Hydrothermal synthesis of nitrogen-doped carbon dot from glucose. High-resolution XPS spectra of (b) N 1s, (c) C 1s, and (d) O 1s. (e) Raman spectrum of the nitrogen-doped carbon dots. (f) Excitation and (g) wavelength-dependent emission spectra. [Reprinted from Tong-Yang Shen, Pei-Yun Jia, Da-Shu Chen, Li-Na Wang, Hydrothermal synthesis of N-doped carbon quantum dots and their application in ion-detection and cell-imaging, *Spectrochimica Acta Part A: Molecular and Biomolecular Spectroscopy*, **248**, 119282, copyright (2021), with permission from Elsevier].^33^

In 2021, Shen *et al.* developed nitrogen carbon quantum dots (N-CQDs) by bottom-up approaches, involving the nitrogenization of CQDs or fusion of carbon precursor in the presence of nitrogenization agent (Fig. 3).^33^ Glucose was utilized as the carbon source and *m*-phenylenediamine served as the nitrogenization agent. Upon dispersing in deionized water under ultrasonication, the precursors were mixed in a 3 : 2 ratio and reacted hydrothermally in a sealed autoclave at 190 °C under high pressure. After 12 h, the N-CQDs were isolated by centrifugation. The N-CQDs were characterized by X-ray photoelectron spectroscopy (XPS), X-ray diffraction (XRD) analysis, Raman spectroscopy, Fourier-transform infrared (FT-IR) spectroscopy, and transmission electron microscopy (TEM), which revealed that N-CQDs contained imine bonds formed between the amine group of the nitrogen precursor and the glucose aldehyde group.

**Fig. 4:**
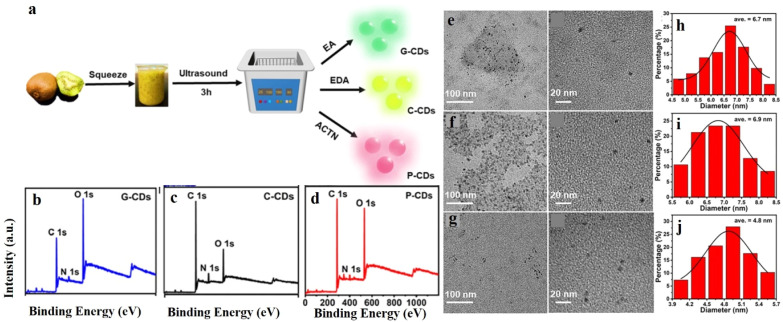
(a) Green synthesis of nitrogen-doped carbon dots *via* ultrasonication. High-resolution XPS full survey of (b) G-CDs, (c) C-CDs, and (d) P-CDs. TEM images of (e) G-CDS, (f) C-CDs, and (g) P-CDs. Results of dynamic light scattering study of (h) G-CDs, (i) C-CDs, and (j) P-CDs. [Reprinted from J. Xu, K. Cui, T. Gong, J. Zhang, Z. Zhai, L. Hou, F. uz Zaman, C. Yuan, Ultrasonic-assisted synthesis of N-doped, multicolor carbon dots toward fluorescent inks, fluorescence sensors, and logic gate operations, *Nanomaterials*, **12**, 312, copyright (2022), with permission from MDPI].^34^

In another recent attempt, Xu *et al.* developed multi-luminescent carbon dots from kiwi fruit by a simple ultrasonication method in the presence of different additives (Fig. 4).^34^ The principle behind the synthesis was the ultrasonication-induced sudden breakdown of the small vacuum bubbles formed, leading to deagglomeration. Such a green mode of synthesis of NCDs is considered highly advantageous. Filtered kiwifruit juice was mixed with the additives in 10 : 1 v/v ratio, followed by treatment with high-intensity ultrasonic irradiation (320 W, 80 kHz) at room temperature for 3 h. The resultant solution was centrifuged, filtered, and purified by dialysis to remove unreacted residues. The formation of NCDs was confirmed by the presence of the N 1s peak in XPS and C=N peaks in the IR spectrum. Variations in morphology and photophysical properties were monitored by using different additives like ethanol, ethylenediamine, and acetone. Among the different types of NCDs produced, the ethylenediamine-derived ones showed pale yellow color.

Otten and co-workers employed pyrolysis and solvothermal methods to prepare NCDs using urea as the nitrogen precursor and citric acid as the carbon source and compared the properties of NCDs obtained under different reaction conditions.^35^ After heating the two precursors in a molar ratio of 0.56 in an autoclave for 20 h and 190 °C, the reaction was quenched and the NCDs were collected as a dispersion in excess water. Thereafter, the NCDs were separated according to their sizes using a dialysis bag. Otten *et al.* established that the presence of urea improved the fluorescence quantum yield of CDs.

**Fig. 5:**
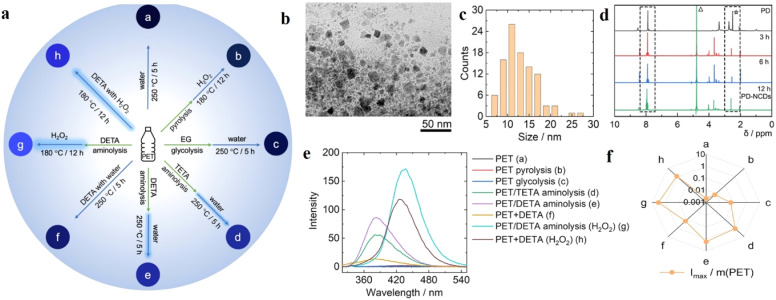
(a) Preparation of different types of carbon dots from polyethylene terephthalate (PET) bottles following diverse reaction methods. (b) TEM and (c) DLS studies for the characterization of the carbon dots, (d) comparative ^1^H NMR spectra of the carbon dots obtained from aminolysis and hydrothermal methods, (e) comparative fluorescence spectra for carbon dots obtained from different methods, and (f) comparative fluorescence intensities of the carbon dots prepared by different methods with respect to the weight of PET consumed. [Reprinted from Kayee Chan, Anatoly Zinchenko, Aminolysis-assisted hydrothermal conversion of waste PET plastic to N-doped carbon dots with markedly enhanced fluorescence, *Journal of Environmental Chemical Engineering*, **10**, 107749, copyright (2022), with permission from Elsevier].^44^

Another prominent route to synthesizing NCDs involves aminolysis. Recently, Chan *et al.* have prepared highly luminescent NCDs from polyethylene terephthalate (PET) derived from waste plastic bottles in the presence of diethylenetriamine (DETA) and triethylenetetramine (TETA) as the nitrogen sources (Fig. 5).^44^ PET powder was refluxed at ∼200 °C with the nitrogen precursor in 1 : 1 molar ratio in the presence of zinc acetate as a catalyst. The aminolysis product was further subjected to hydrothermal heating overnight in a Teflon autoclave in the presence of an oxidizing agent (H_2_O_2_) in 1 : 1 w/v ratio. They also compared the optical properties of the CDs synthesized from PET following other methods like glycolysis, pyrolysis, *etc.* and found that the aminolysis products showed high luminescence with a quantum yield of 9.1%. This can be attributed to the higher degree of functionalization for the aminolysis-derived NCDs, probably due to the depolymerization of PET and formation of amidoamines in the presence of DETA or TETA.

In addition to the versatile methods discussed above, there are several other prominent methods by which NCDs with excellent luminescence properties have been synthesized. Microwave irradiation is one among such cost-effective modes of preparation for NCDs; microwave-prepared NCDs displayed fluorescence quantum yield as high as 73%. Besides nitrogen, there are several non-metals like boron, sulfur, *etc.*, which have been utilized as doping agent for CDs (Table 1). However, NCDs exhibited the best results owing to structural compatibility between nitrogen and carbon.

**Table 1:** Examples of other non-metal-doped carbon dots in fluorescence sensing

SI. no.	Type of doping	Method of synthesis	Biomarker	Limit of detection (LOD)	Application	References
1	Nitrogen doping	One-pot solid phase pyrolysis	Human serum albumin	Binding constant: 3.105 × 10^4^ L mol^−1^	Fluorescence quenching due to the formation of ground state complex between CDs and human serum albumin *via* hydrophobic interactions and hydrogen bonding	45
2	Hydrophilic nitrogen doping	Hydrothermal process	Fe^3+^	0.66 µM	Fluorescence quenching-mediated detection of Fe^3+^*via* charge transfer mechanism	46
3	Nitrogen doping	Hydrothermal method	Bilirubin, vitamin B_12_	56.28 nM for B_12_ and 89.07 nM for bilirubin	Fluorescence quenching for B_12_*via* inner filter effect; whereas bilirubin served as an efficient FRET acceptor with respect to the donor, CDs leading to quenching of CD fluorescence	47
4	Phosphorus doping	Hydrothermal method	Fe^3+^	9.5 nM	Static fluorescence quenching due to formation of ground state chelate complex between CD and Fe^3+^	48
5	Nitrogen and sulfur co-doping	Hydrothermal method	Cu^2+^, Pb^2+^, temperature	200 µM	Dynamic fluorescence quenching in presence of Cu^2+^ and Pb^2+^ due to ultrafast electron transfer. These CDs also showed fluorescence quenching at higher temperature owing to increase in the non-radiative surface states	49
6	Nitrogen and fluorine co-doping	One-step microwave-assisted technique	Fe^3+^, ascorbic acid	1.03 µM for Fe^3+^, 4.22 µM for ascorbic acid	Static fluorescence quenching due to formation of ground state chelate complex between CD and Fe^3+^ and inner filter effect-induced quenching in presence of ascorbic acid	50
7	Nitrogen and sulfur co-doping	Microwave pyrolysis	Glutathione	30.6 mM for Cu^2+^, 3.74 µM for glutathione	Fluorescence switch “on–off–on” response upon periodic addition of Cu^2+^ and glutathione due to inner filter effect	51
8	Nitrogen, sulfur, and phosphorous co-doping	Microwave-induced pyrolysis	Cr(vi)	4 nM	Fluorescence quenching due to inner filter effect, which is ascribed to strong overlap between the absorption of Cr(vi) with the excitation of CDs	52
9	Nitrogen, sulfur, and iodine co-doping	Hydrothermal method	Folic acid	84 nM	Fluorescence change from blue to green on hydrogen bonding with folic acid facilitating FRET the CDs to folic acid	53
10	Boron and nitrogen co-doping	One-pot hydrothermal method	Cu^2+^	1.6 nM	Fluorescence quenching on complexation with Cu^2+^ facilitating electron transfer from the CDs to the vacant d-orbital of Cu^2+^	54

#### Metal-doped carbon dots

Another class of CDs are the metal-doped ones. Metallic doping agents were found to influence the characteristic physicochemical properties of CDs to a greater extent than non-metallic ones. This can be ascribed to the availability of electrons and vacant orbitals in the metals, which allows the dopant metals to undergo diverse energy transfer processes, leading to changes in the HOMO–LUMO energy gap of the CD structure. Therefore, such metal-doped CDs have shown highly promising optical properties. Most significant metals used for doping CDs are transition metals like copper (Cu),^55,56^ manganese (Mn),^57,58^ silver (Ag),^59^ magnesium (Mg),^60^ gadolinium (Gd),^61^ zinc (Zn),^62^ cobalt (Co),^63^*etc.*

Doping with metals have been known to improve the stability, water solubility, and biocompatibility of CDs. However, excess metal doping can prove to be toxic under physiological conditions. Therefore, the ratio of carbon precursor to metal dopant should be optimized very critically.

**Fig. 6:**
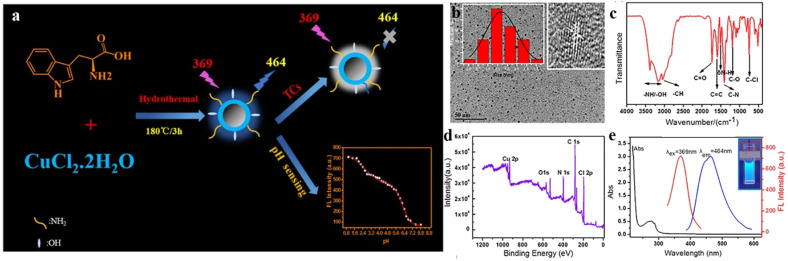
(a) Schematic of the synthesis of Cu-doped carbon dots (Cu-CDs). (b) TEM image, (c) IR spectrum, and (d) XPS spectrum of the Cu-CDs. (e) Excitation and emission spectra of the Cu-CDs. [Reprinted from Jianhua Guo, Wenjing Lu, Huilin Zhang, Yating Meng, Fangfang Du, Shaomin Shuang, Chuan Dong, Copper doped carbon dots as the multi-functional fluorescent sensing platform for tetracyclines and pH, *Sensors and Actuators B: Chemical*, **330**, 129360, copyright (2021), with permission from Elsevier].^36^

Most metal-doped CDs have shown improved catalytic application. However, sensing of different biomarkers is the need of the hour for the advancement of biomedical applications of CDs. In this regard, Guo *et al.* constructed copper-doped CDs (Cu-CDs) for the selective detection of tetracycline and pH of the cellular microenvironment (Fig. 6).^36^ They synthesized Cu-CDs following hydrothermal heating of the carbon source tryptophan with CuCl_2_·2H_2_O in 1 : 2 molar ratio at 180 °C for 3 h in an autoclave. The dots were then isolated by filtration using filter membrane and dialysis, followed by freeze-drying. Thus-obtained CDs were of spherical in shape with a diameter as small as 4.84 nm. The fluorescence intensity of the CDs was found to decrease in the presence of tetracycline due to inner filter effect.

**Fig. 7:**
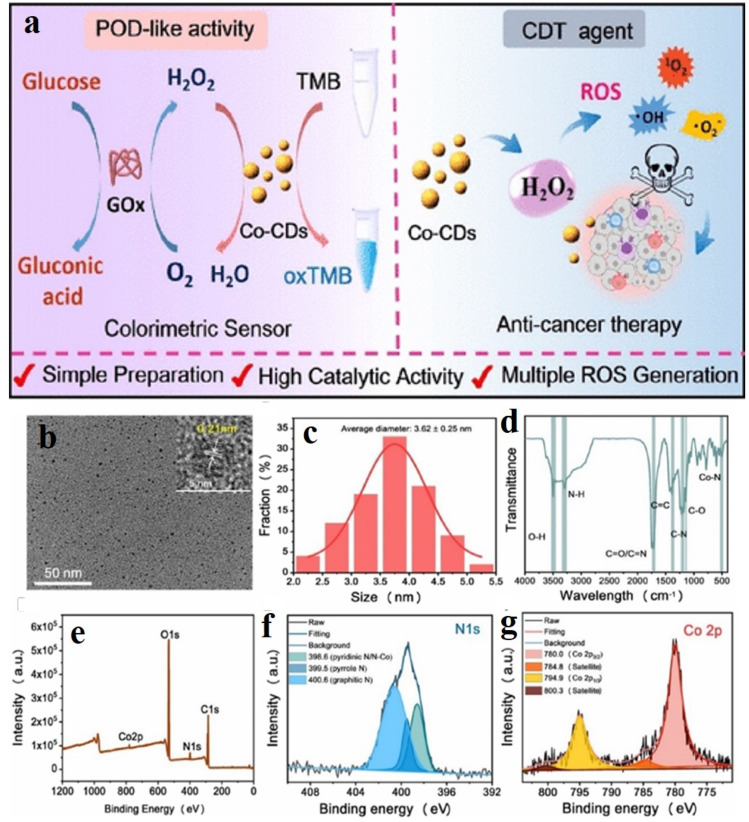
(a) Schematic of the glucose sensing mechanism by Co-doped carbon dots (Co-CDs). (b) TEM image of Co-CDs, (c) dynamic light scattering for size determination of carbon dots. (d) IR and (e) XPS spectra for the characterization of the Co-CDs. High-resolution XPS of (f) N 1s and (g) Co ^2^p_3/2_. [Reprinted with permission from *ACS Appl. Mater. Interfaces*, 2022, **14**(51), 57206–57214. Copyright 2022 American Chemical Society].^37^

In a recent study by Lu *et al.*, cobalt-doped CDs (Co-CDs) for synergistic sensing, enzymatic, and catalytic activities^37^ were prepared from green cobalt and carbon precursors like vitamin B_12_ and citric acid by a pyrolysis method. Heating the precursors at 180 °C afforded a gelatinous product, which was isolated upon further heating and centrifugation. The cobalt center in vitamin B_12_ served as the active site for the enzyme glucose peroxidase, which could convert glucose into gluconic acid and H_2_O_2_. Owing to the polyvalence of cobalt, it can show prominent redox properties. Therefore, the as-synthesized Co-CDs were able to oxidize 3,3′,5,5′-tetramethylbenzidine (TMB) in the presence of H_2_O_2_, which helped in quantifying the amount of glucose converted into H_2_O_2_ (Fig. 7).^37^ The Co-CDs also produced several reactive oxygen species like hydroxyl radical, superoxide ion, singlet oxygen, *etc.* upon oxidation of H_2_O_2_ to achieve successful chemodynamic treatment in cancer cells.

**Fig. 8:**
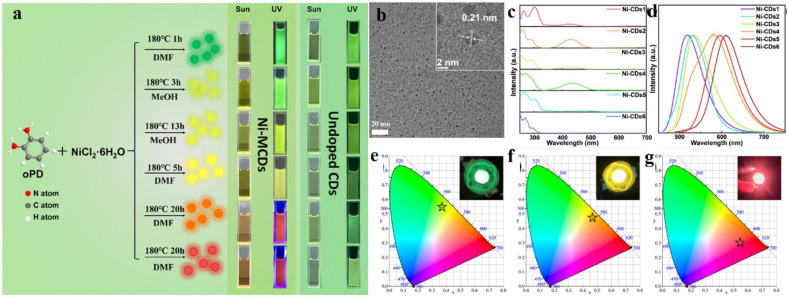
(a) Schematic of the synthesis of diverse multi-colored Ni-doped carbon dots. (b) Representative TEM image of the carbon dots obtained after 1 h of reaction. (c) Absorption and (d) emission spectra of different carbon dots, (e–g) CIE coordinates of the different types of Ni-doped carbon dots. [Reprinted from Yunhui Wu, Jiurong Li, Xiujian Zhao, Xiao Gong, Nickel-doped carbon dots with enhanced and tunable multicolor fluorescence emission for multicolor light-emitting diodes, *Carbon*, **201**, 796–804, copyright (2023), with permission from Elsevier].^64^

Wu *et al.* recently developed a series of multi-color, multi-sized nickel-doped CDs (Ni-CDs; Fig. 8),^64^ by heating the carbon precursor, *viz.*, *o*-phenylenediamine, with NiCl_2_⸱6H_2_O at 180 °C in an autoclave for different durations in the presence of different solvents. With increase in reaction time, both the size and fluorescence quantum yield of the Ni-CDs increase. The fluorescence color for the Ni-CDs was extended as far as red, while non-doped CDs showed fluorescence only up to the yellow region. Such shift in the emission spectrum was attributed to the different surface functionalities and different reaction times. Different reaction times induce different HOMO–LUMO energy gaps, which also contributed to the shift in the emission spectra.

Various other examples of metal doping on carbon dots have been reported, which have proved useful for biomedical applications. Magnesium-doped CDs (Mg-CDs) were developed for application in bone regeneration.^65^ For this class of CDs, the weight ratio of the carbon precursor and the magnesium salt was optimized as 1 : 4 under hydrothermal reaction conditions. These Mg-CDs displayed significant *in vitro* differentiation and proliferation of osteoblasts. Some metal-doped CDs have found application in metal ion sensing and other catalytic activities.^66^

The methodologies for synthesizing carbon dots differ significantly in complexity, scalability, and environmental impact, each presenting unique benefits and drawbacks. Hydrothermal techniques are highly favored for their superior control over particle size and surface chemistry, exceptional biocompatibility, and ease of heteroatom doping. Nonetheless, they necessitate extended reaction durations (3–12 hours), elevated temperatures (120–220 °C), and post-synthesis purification, which may lead to batch variability.^67^ Green hydrothermal techniques utilizing biomass precursors enhance sustainability and cost-efficiency while preserving biocompatibility. These techniques are labor-intensive and susceptible to feedstock fluctuation.^68^ Microwave-assisted synthesis provides expedited processing (≤10 min), energy efficiency, and scalability, yielding carbon dots with favorable optical properties. However, it poses concerns of uneven heating and necessitates specialized reactors.^69,70^ Microwave pyrolysis similarly expedites carbonization and facilitates doping, although offers diminished control over particle size and shape.^71^ Thermal pyrolysis is straightforward and scalable, yielding highly stable carbon dots, but it requires high temperatures. Graphitization can be accomplished with constrained functionalization.^72^ Acidic oxidation coupled with hydrothermal treatment produces highly functionalized carbon dots abundant in –COOH groups, which improves enzyme immobilization and surface regulation. These protocols entail intricate, time-consuming procedures and acid management.^73^ Electrochemical synthesis and exfoliation are distinguished by their simplicity, mild conditions, and scalability, yielding carbon dots with adjustable size and surface functional groups. However, they frequently necessitate ionic liquids or particular electrolytes.^74,75^ Alternative emerging methodologies such as acid digestion, solvent-free mortar-pestle heating, solvothermal pyrolysis, ultrasonication-induced synthesis, and hybrid techniques like aminolysis combined with hydrothermal oxidation or glycolysis present specific advantages in terms of simplicity or functionalization, yet frequently exhibit deficiencies in reproducibility and scalability.^76^

## Harnessing carbon dots for advanced biosensing

Carbon dots have been widely used in the field of biosensing owing to their intriguing optical properties, biocompatibility, and cellular accumulation. By virtue of their excellent fluorescence emission and long-range absorption up to the near-infrared (NIR) region, CDs have long been a handy choice for the diagnosis of diverse diseases. Although there are different hypotheses for the source of fluorescence in CDs, the most suitable and widely accepted one is the sp^2^-carbon core emission theory.^77^ The CDs synthesized from different sources by applying different methods constitute an aromatic graphitic sp^2^-core structure, within which π–π* transitions lead to extended absorption to longer wavelengths.^78^ Introduction of a dopant in the CD structure often induced a red-shift the absorption spectrum, owing to the dopants incorporating new defect bands within the existing HOMO and LUMO levels, thus reducing the energy gap.^79^ One more way to tune the optical properties of the CDs involves varying the surface functionalization. Surface functionalities give rise to newer π–π* or *n*–π* transitions, thereby shifting both absorption and emission wavelengths. There have been many studies that have elaborated the sources of optical properties of CDs.^80–82^

The significance of CDs as biosensors lie in their specificity and environment-sensitive tunable emission properties. The emission properties of the carbon dots can be modulated very easily by virtue of their ability to function both as electron donor and electron acceptor. Such electron transfer ability of CDs enable them to participate in various energy/charge transfer processes, *viz.*, fluorescence resonance energy transfer (FRET), internal charge transfer (ICT), photoinduced electron transfer (PET), aggregation-induced emission (AIE), *etc.*, which regulate the fluorescence properties of any probe.^81–84^ The energy/charge transfer processes can be facilitated by suitably decorating the surfaces of CDs with potential fluorophores. The enhanced specificity of CDs in biosensing and other treatment modalities is often achieved by introducing suitable surface functionalities. Target-specific treatment is a primary concern in terms of precise point-of-care therapy; the ability of CDs to target biomolecules like oligonucleotides, aptamers, antibodies, *etc.* help in this regard. The following section aims to shed some light on the two important criteria to devise CDs as efficient biosensors: (i) modulation of electronic properties and (ii) introducing target biomolecules as surface functionality.

### Modulating fluorescence properties

**Fig. 9:**
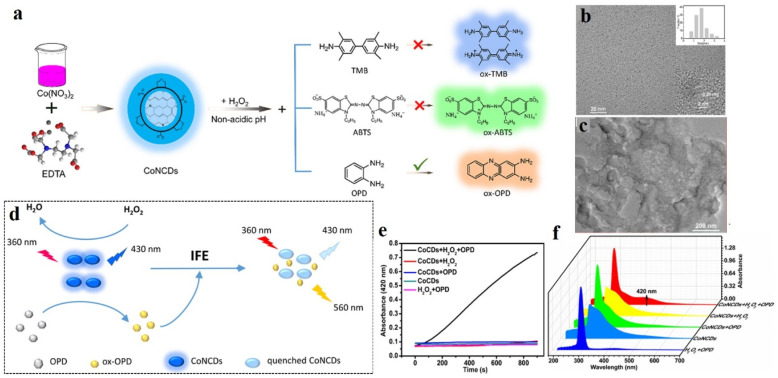
(a) Preparation of Co-doped carbon dots for peroxidase-like catalytic properties. TEM images (b) before and (c) after using the carbon dots as catalysts. (d) Probable mechanism of hydrogen peroxide sensing. (e) Absorption and (f) emission spectra of Co-doped carbon dots under different conditions. [Reprinted from Li Su, Sainan Qin,Yexi Cai, Liang Wang, Wenpei Dong, Guojiang Mao, Suling Feng, Zhongjian Xie, Han Zhang, Co, N-doped carbon dot nanozymes with acid pH-independence and substrate selectivity for biosensing and bioimaging, *Sensors and Actuators B: Chemical*, **353**, 131150, copyright (2022), with permission from Elsevier].^86^

Attributed to their sp^2^-carbon core and their wide and excitation-wavelength-dependent emission properties, CDs often act as energy/charge donors. Mostly, CDs are synthesized by the aggregation of carbon-rich sources, which makes the CD core structure highly electron-rich. Functionalization with suitable electron-accepting groups can facilitate charge transfer processes, leading to abrupt changes in the electronic arrangement in the CD structure, thus altering their fluorescence properties. One of the recent examples involve decorating CDs with electron-withdrawing 2,3-dicyanohydroquinone, affording red-emissive supra-carbon dots.^85^ In another study, Su *et al.* prepared carbon-dot-based nanozymes following a hydrothermal method from ethylene diamine tetraacetic acid disodium salt and Co(NO_3_)_2_·6H_2_O (Fig. 9) and used the same for successful detection of cholesterol, xanthine, and hydrogen peroxide.^86^ The Co- and N-doped carbon dot nanozymes exhibited excellent peroxidase activities, as confirmed by EPR spectroscopic studies, which revealed the formation of hydroxyl radical from H_2_O_2_*via* the ping-pong mechanism. Further, these nanozymes showed prominent colorimetric and ratiometric changes in their absorption and emission properties in response to H_2_O_2_ and cholesterol. The mechanism of H_2_O_2_ detection by the oxidation of *o*-phenylenediamine (OPD) was established. The oxidized form of *o*-phenylenediamine (OxOPD) could efficiently quench the inherent emission of CDs *via* inner filter effect. The emission at 560 nm was found to gradually increase with increasing cholesterol concentration, which was quantified in terms of the H_2_O_2_ produced by the oxidation of cholesterol.

**Fig. 10:**
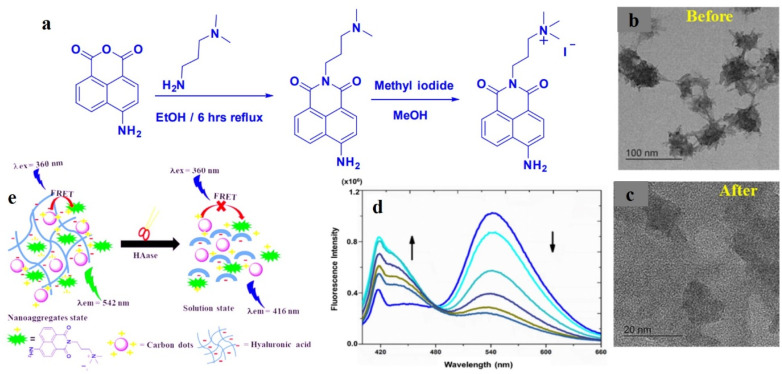
(a) Synthesis of the naphthalimide-based fluorescent probe. TEM images of naphthalimide-decorated carbon dots (b) before and (c) after treatment with hyaluronidase. (d) Change in emission spectra upon gradual addition of hyaluronidase. (e) Probable mechanism of FRET-based sensing of hyaluronidase by naphthalimide-functionalized carbon dots. [Reprinted from P. Raj, S. Lee, T. Y. Lee, Carbon dot/naphthalimide based ratiometric fluorescence biosensor for hyaluronidase detection, *Materials*, **14**, 1313, copyright (2021), with permission from MDPI].^88^

Similarly, CD nanoparticles, synthesized from *o*-phenylenediamine, were utilized for the biosensing of Cu^2+^ and glutathione.^87^ In this case, OPD underwent oxidation in the presence of Cu^2+^, and excellent overlap was observed between the emission spectrum of CDs (*λ*_em_ = 446 nm) and the absorption spectrum of oxidized OPD (*λ*_abs_ = 416 nm). Such strong spectral overlap induced facile energy transfer from the donor, *i.e.*, the CDs to oxidized OPD, thus quenching the characteristic fluorescence of CDs. Raj *et al.* prepared a supramolecular nanoassembly combining CDs and a naphthalimide probe for the efficient sensing of hyaluronidase enzyme.^88^ In this case, negatively charged CDs were adsorbed on the surface of hyaluronic acid, along with a positively charged naphthalimide probe. The close proximity of the CD and the probe and the spectral overlap between them facilitated a strong FRET process from the CDs to naphthalimide, which is exhibited by the characteristic green fluorescence of the naphthalimide moiety (Fig. 10). Upon interaction with hyaluronidase enzyme the supramolecular network got disrupted, thereby ceasing the FRET process. As a result, the inherent blue fluorescence of the CDs was restored.

**Fig. 11:**
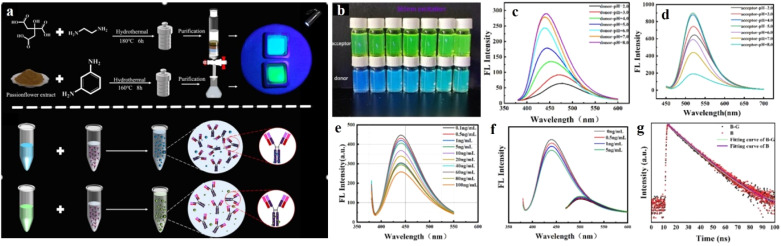
(a) Synthesis of donor carbon dots and acceptor carbon dots, followed by functionalization with anti-procalcitonin antibodies. (b) Photographic images of both donor and acceptor carbon dots under UV lamp. Fluorescence spectra of the (c) donor and (d) acceptor carbon dots under different pH conditions, and of (e) antibody-functionalized donor carbon dots in the presence of procalcitonin and (f) the mixture of antibody-functionalized donor and acceptor carbon dots in the presence of procalcitonin. (g) Change in fluorescence lifetime of both donor and acceptor carbon dots on interaction with procalcitonin. [Reprinted from Yuxin Tian, Yuwei Du, Zhang Chen, Li Li, Ruhong Yan, Xiaoqiang Li, Juan Yue, Rapid detection of procalcitonin using dual-color carbon quantum dots as fluorescent probes in FRET assay, *Spectrochimica Acta Part A: Molecular and Biomolecular Spectroscopy*, **338**, 126207, copyright (2025), with permission from Elsevier].^90^

By virtue of their tunable energy gap, CDs can also act as energy-acceptor units despite having electron-rich sp^2^ core. Functionalization with a suitable electron-accepting group can enable CDs to accept energy or charge density. This tunability of the energy gap leads to intriguing optical and electronic properties. Recently, Zhang *et al.* has reported a series of six NCDs prepared from three different nitrogen sources, *viz.*, semicarbazide, urea, and formamide.^89^ Upon varying the reaction conditions and the solvent used, the optical properties of the six NCDs were successfully regulated. The carbon dots were decorated with electron-rich surface functional groups like –NH_2_, –OH, *etc.* Detailed solvent study revealed that the inherent fluorescence of the NCDs originated from the local emission states within the NCD core owing to excited-state intramolecular charge transfer (ESIPT). Under high-pH (basic) conditions, electron transfer from the photoexcited HOMO of the surface functional groups to the LUMO of the electron-accepting carbon core was facilitated, thus quenching the fluorescence of the NCDs. However, under low-pH (acidic) conditions, the LUMO of the protonated surface functionalities remained available for electron acceptance from the carbon core, which in turn further quenched the fluorescence of the NCDs. Thereby, the NCDs synthesized in this study were helpful for monitoring the intracellular pH. Tian *et al.* also prepared two different sets of CDs with blue and green fluorescence properties (Fig. 11).^90^

Two different CDs were synthesized from two different precursors, *viz.* ethylenediamine and *meta*-phenylenediamine, and were utilized for the specific detection of procalcitonin (PCT), a biomarker of post-operative infections. To achieve sensitive detection, both the CDs were decorated with PCT antibodies *via* 1-ethyl-3-(3-dimethylaminopropyl)carbodiimide (EDC) coupling. Increasing concentration of PCT antigens facilitated specific binding with both CDs, bringing them close enough to achieve efficient FRET. The fluorescence spectra showed a prominent shift from blue to green, further establishing the energy transfer from blue CDs to green CDs. Huang *et al.* also showed the electron-accepting property of the CDs in one of their recent findings.^91^ In this study, CDs were utilized for the selective sensing of the organic pollutant tetracycline, which quenched the inherent fluorescence of CDs by the transfer of electrons from the HOMO of the electron-rich donor (tetracycline) to the LUMO of the CDs.

The dual redox property of the CDs makes them useful for biosensing as well as in fields like solar cell, catalysis, light harvesting, *etc.* As illustrated above, the unique redox property of CDs stems from the versatile functionalities on its surface, depending on the precursors used for their synthesis. Besides covalent attachment of the suitable electron donor or acceptor on the surface of carbon dots, non-covalent interactions like π-stacking or electrostatic interaction played pivotal roles in dictating the redox property of CDs. Hydrogen bonding with a suitable electron donor or acceptor moiety can also prove useful in this regard owing to the strong directional nature of such bonds. Hence, new surface functionalities can be explored by varying the interaction method to achieve better electron/charge transfer processes.

### Tuning other optical properties

Besides such intriguing photoluminescence properties, other optical properties like chemiluminescence, upconversion luminescence, two-photon absorption, *etc.* also accentuate the suitability of carbon dots for biosensing. CDs have been found to exhibit efficient two-photon absorption ability.^92–94^ The two-photon absorption especially observed in case of heteroatom-doped CDs can be ascribed to the insertion of new energy levels within the original HOMO and LUMO resulting in reduced band gap and facile transition from the non-bonding orbital of the heteroatom to the π* orbital of CD core.^95,96^ Two-photon absorption involves excitation of an electron from the ground state to first excited singlet state following a concurrent absorption of two photons with half energy and double the wavelength with respect to the band gap.^97^ Owing to the non-linearity of two-photon absorption, the out-of-focus fluorescence emission can be avoided to a great extent. The two-photon absorption of CDs is of particular interest for biosensing purpose as it involves absorption of longer wavelength even up to near infrared (NIR) region. Suitable designing of two-photon absorbing CDs include combination of appropriate donor and acceptor *via* conjugated π-linker. Symmetric structure with strong donor/acceptor pairs can induce higher two-photon absorption cross-section in CDs even at a very low concentration. Such donor/acceptor pairs results in stronger intramolecular charge transfer (ICT). A recent work by Lesani and colleagues highlighted a fluorescein isothiocyanate (FITC)-conjugated nitrogen-doped CDs for fascinating dual emission and highly sensitive detection of Fe^3+^.^93^ Conjugation with FITC increased the π-electron density, thereby reducing the π–π* band gap. Further, FITC-CDs showed excellent dual emission properties at 460 nm and 520 nm originating from CD core and FITC, respectively. With increasing nitrogen-doping, surface defects in CDs also increases proportionally. This was marked by the preparation of a *o*-phenylenediamine-derived Mn-doped CDs with improved, red-shifted dual emission property.^98^ The two-photon absorption of CDs facilitates emission at a shorter wavelength with respect to excitation wavelength.^99,100^ Such anti-Stokes shift induces upconverting fluorescence emission in the CDs. Upconversion fluorescence is more effective in terms of biosensing compared to regular fluorescence emission attributing to their deep tissue penetration and high signal to noise ratio. Size, morphology, surface functional groups have been found to have large impact on regulating the upconversion emission in CDs.^99^ In a recent work, Lu *et al.* reported a blue emitting CD from citric acid and *o*-phenylenediamine in presence of sunlight.^101^ In this case, a strong excitation wavelength was observed at 750 nm with an emission wavelength at 462 nm supporting captivating upconversion effect. This upconversion property stemmed from increased amount of electronegative nitrogen atom at the core of the CDs. The higher electron density of nitrogen atoms can bring about dissimilarity in the excited state electronic levels leading to multiple equilibrium states. The emission of the CDs was greatly quenched by intracellular Hg^2+^ owing to aggregation of CDs leading to static quenching. Another report from Gogoi *et al.* utilized the upconverted NIR emission of the CDs for successful biosensing of glutathione.^102^ These CDs were synthesized by hydrothermal method from starch and citric acid. These CDs displayed excellent anti-Stokes shift of greater than 300 nm with a strong emission in the green region around 520–540 nm. The upconversion of fluorescence, in this case, originated from the multiple surface states present in the CDs, which could act as emission traps. To monitor the sensing ability the CDs were first treated with a solution of Cu^2+^, which showed strong interaction with the emission traps leading to quenching of the green fluorescence. However, addition of successive amount of glutathione replaced the Cu^2+^ ions from the emission traps restoring the inherent fluorescence of the CDs. Of late, much research has been done to explore different doping to improve the upconversion fluorescence of CDs, *e.g.*, lanthanide atoms, TiO_2_, *etc.*^103,104^ Doping with these materials creates higher defect sites on the CD surface and improves two-photon absorption.

Another interesting optical property of CDs is chemiluminescence. Chemiluminescence is a method, where the electron gets excited by means of a chemical reaction rather than absorption of light. As discussed earlier, CDs can serve both as efficient electron donors and acceptors by virtue of their electron rich sp^2^-core and presence of suitable dopants. Exploiting this intriguing property of CDs, many research groups have developed biosensors based on chemiluminescence. One of the recent findings by Shokri and his team showcased a B, N-doped CD for selective detection of antibiotic, rifampicin.^105^ Doping with B and N accelerated the electron–hole recombination reactions in the CDs leading to higher quantum yield values. The absorption spectra the CDs exhibited strong overlap with the emission of electron donating SO_2_* in a Ce(iv)–SO_3_^−^ chemiluminescence system. These CDs were utilised to monitor the rifampicin assay in biological system. Yang *et al.* Developed another class of CDs doped with chlorine and phosphorous.^106^ The presence of chlorine makes the CDs efficient energy acceptor, which could show profound catalytic activity. These CDs could initiate the degradation of H_2_O_2_ in a H_2_O_2_–KMnO_4_ chemiluminescence system resulting in formation of reactive oxygen species like singlet oxygen superoxide ion, *etc.* On oxidation by the reactive oxygen species, the CDs reached the excited state. In presence of Cu^2+^, the H_2_O_2_ degraded to a greater extent leading to formation of more reactive oxygen species, which in turn increases the chemiluminescence of the CDs. Following this line of research Cu^2+^-doped CDs have been utilized to detect glucose levels in the physiological systems exploiting the catalytic behaviour of Cu-CDs in presence of H_2_O_2_.^107^ Upon gradual addition of glucose, H_2_O_2_-mediated oxidation of Cu-CDs produced excellent chemiluminescence signal at 425 nm. However, lower quantum yield, reaction kinetics-dependent emission intensity and duration, critical reaction mechanism *via* multistep reactive intermediate generation, *etc.* restricts the application of chemiluminescence in the field of biosensing. Attributing to their extraordinary electron donating and accepting properties, CDs are also capable of exhibiting electrochemiluminescence property.^108^ This method mostly involves grafting of biomolecules like antibodies, aptamers, enzymes, *etc.* on electrodes for selective detection of biomarkers related terminal diseases like cancer *via* redox reactions. This method is beyond the scope of this article.

### Surface modification with biomolecules

As mentioned in the previous section, CDs have been extensively utilized for biosensing. However, the shortcomings of some of the biosensing mechanisms include lack of bioavailability, solubility, and poor target specificity. One handy way to address these issues involves decorating the CDs with targeting biomolecules like antibody, aptamers, DNA, *etc.* By virtue of the specific interactions of the biomolecules within the physiological environment, such CDs show extraordinary biocompatibility and precise interaction with the target bio-analyte. This precise interaction induces prominent changes in the emission properties of the CDs, facilitating efficient biosensing.

### Antibody-functionalized carbon dots

Antibodies are a class of proteins generated from the immune system of most vertebrates.^109,110^ These Y-shaped immunoglobulins containing heavy- and light-chain amino acid sequences mostly connected *via* disulfide bonds can interact selectively with epitopes of antigens mainly *via* strong covalent interactions.^111^ Therefore, monitoring antigen–antibody interaction is considered one of the promising methods of biosensing. By virtue of the large number of active sites on CD surfaces, they are prone to versatile functionalization. Functionalization of CDs with antibodies will render them suitable for the sensing of diverse biomolecules, *e.g.*, few specific virus, bacteria, or protein molecules, which do not react directly with usual CD-based biosensors.

**Fig. 12:**
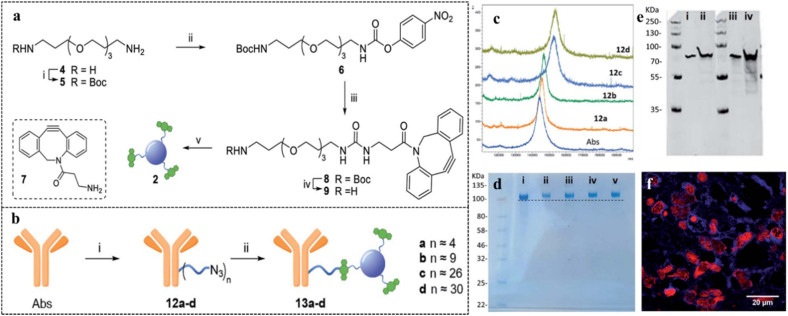
(a) Preparation of dibenzocyclooctyne-functionalized CDs. (b) Conjugation of antibodies on the surface of CDs *via* amide bond formation. (c) Matrix-assisted laser desorption/ionization time-of-flight (MALDI-TOF) analysis of antibody-decorated CDs, (d) NuPAGE gel electrophoresis for different antibody derivatives: (i) native antibody, (ii) 13a, (iii) 13b, (iv) 13c, and (v) 13d. (e) Western blot studies with recombinant human GFAP protein and antibody-CD composite, 13a. (f) Immunostained tissue section of malignant brain tumor glioblastoma with antibody-CD composite, 13a, red-labeled nuclei and blue-labeled cobweb pattern of intermediate filament GFAP. [Reprinted from M. Ghirardello, R. Shyam, X. Liu, T. Garcia-Millan, I. Sittel, J. Ramos-Soriano, K. M. Kurian, M. C. Galan, Carbon dot-based fluorescent antibody nanoprobes as brain tumour glioblastoma diagnostics, *Nanoscale Adv.*, **4**, 1770–1778, copyright (2022), with permission from Royal Society of Chemistry].^112^

Brain cancer is one of the most difficult forms of cancer to diagnose owing to the presence of the blood–brain barrier, which prevents penetration of the probes to the brain. There are several biomarkers related to brain cancer that are soluble in peripheral blood. Glial fibrillary acid protein (GFAP) is one of the prominent biomarkers for brain cancer.^112^ Specific antibodies have been found to easily permeate through the blood–brain barrier. Recently, Ghirardello *et al.* made use of the permeability and specificity of antibody-decorated CDs for the timely diagnosis of brain cancer (Fig. 12).^112^

Dibenzocyclooctyne-functionalized CDs were synthesized starting from citric acid and ethylene diamine by a microwave method. Azide-functionalized anti-GFAP antibodies were loaded on the CD surface *via* amide bond formation. Bright-blue fluorescence was observed in the confocal microscopic studies, which implied that the antibody-conjugated CDs successfully stained the GFAP intermediate filament in the glioblastoma cell cytoplasm. In another study by Deb *et al.*,^113^ a human immunoglobulin-G-decorated CD was synthesized from orange juice by a green microwave method. The thus-synthesized CDs were utilized for the fluorescence-based detection of vascular endothelial growth factor (VEGF), an angiogenesis biomarker, exploiting an antibody–antigen–antibody sandwich mechanism. The sandwich system was achieved *via* co-incubation of detector antibody-decorated carbon dot, the antigen, VEGF, and a VEGF-specific capture antibody. Upon formation of a complex between VEGF and the antibody, the fluorescence intensity increased more than two-fold, with a detection limit of 5.65 pg mL^−1^. This increment in fluorescence intensity was attributed to the radiative electron–hole pair recombination on the CD surface. Formation of the VEGF-antibody complex induced surface passivation on the CDs, producing more surface vacancies and reducing the surface trap states. The increased number of surface vacancies facilitated rapid electron–hole recombination. A similar sandwich mechanism was reported by Han *et al.*, who utilized silver nanoparticles (Ag NPs) to improve the emission signal of CDs with respect to human epididymis protein 4 (HE 4), an ovarian cancer biomarker owing to their localized surface plasmon.^114^ The CDs were synthesized from folic acid and ethylene glycol by a hydrothermal method. It was further functionalized with capture antibody for HE 4 *via* EDC coupling. On the other hand, Ag NPs were decorated with detection antibody *via* covalent and electrostatic interactions. When a mixture of CD-capture antibody composite and Ag NP-detector antibody composite came in contact with the HE 4 proteins, the capture antibody formed a specific complex with the protein. Such complexation led to antibody–antigen–antibody sandwich aggregation, which in turn brought the CD and the Ag NPs closer to each other. Owing to such close proximity and the strong overlap of the absorption spectra of Ag NPs with the emission spectra of CDs, smooth metal-enhanced emission was observed between the CDs and the Ag NPs, thus improving the fluorescence intensity by a factor of almost 2.1. Hence, the antibody-decorated CD-Ag NPs combination could be successfully used as a turn-on point of care tool for cancer diagnosis.

### Aptamer-functionalized carbon dots

**Fig. 13:**
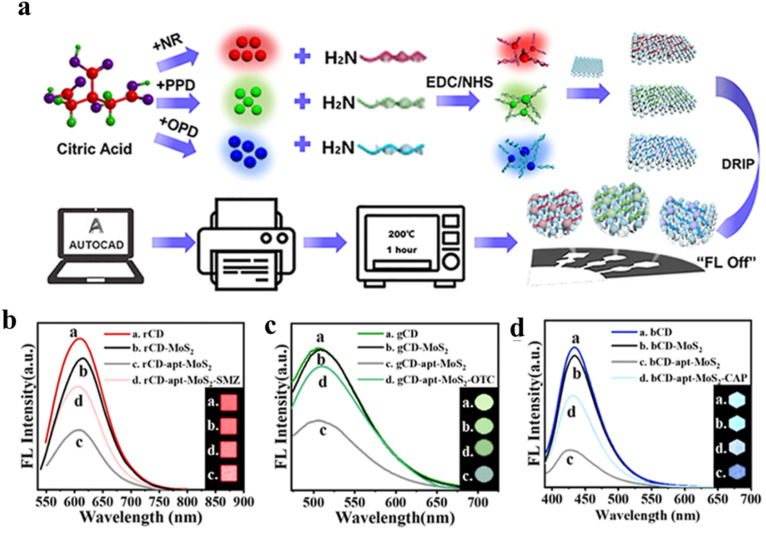
(a) Preparation of microfluidic chip based on red, green, and blue CDs. Fluorescence sensing of (b) sulfamethazine (SMZ), (c) oxytetracycline (OTC), and (d) chloramphenicol (CAP) by red, green, and blue CDs. [Reprinted with permission from *ACS Sens.*, 2022, **7**(12), 3947–3955, copyright 2022 American Chemical Society].^117^

Aptamers are a class of oligonucleotides with a single-strand DNA or RNA structure and short chain length. By virtue of the specific sequence in their strands, aptamers have shown specific interactions with biological targets,^115^ along with biostability, cost-effectiveness, and robust sensing ability.^115^ Contrary to antibodies, aptamers interact with targeted biomolecules *via* hydrogen bonding and other van der Waals forces. Clubbing the specificity and biostability of aptamers with the intriguing optical properties of CDs can afford an array of proficient biosensors. Making use of this idea, Rahmatian and co-workers developed a chitosan-carbon dot composite decorated with a specific aptamer on its surface,^116^ and monitored the infectious disease, Leishmaniasis, by selective interaction with the responsible protein, poly(A) binding protein (PABP). The chitosan-carbon dot nanocomposite did not show any prominent fluorescence upon surface functionalization with aptamer, owing to the FRET process between the ring structure of the aptamer, obtained from the electrostatic interaction between aptamer and the CD core, with the surface functional groups on CDs like phosphate, –NH_2_, –OH, *etc.* The aptamer displayed strong binding affinity with the PABP protein *via* hydrogen bonding, thus detaching the aptamer from the CD surface. In the absence of the aptamer on the surface, CD nanocomposites recovered their inherent fluorescence; they showed an excellent detection limit of 94 cells per mL and a very short response time. To advance from the laboratory-based method as discussed above to a practical one, paper-based microchips connected to a smartphone application have been found to be more effective in terms of biosensing. To achieve this, the CDs have been combined with two-dimensional (2D) MoS_2_ nanosheets,^117^ in which MoS_2_ not only served as a good 2D platform facilitating the formation of laser-printed microchips, but also could act as a strong charge acceptor in connection with the CDs. Three different CDs, with red, blue, and green fluorescence, were synthesized and decorated with antibiotic-specific aptamers. Further, the aptamer-CD hybrid was adsorbed on the surface of the 2D MoS_2_ nanosheet. Upon close contact with the MoS_2_, CDs showed prominent FRET with quenching of fluorescence. However, in the presence of specific antibiotic, the aptamer was detached from the nanosheet surface and formed a complex with the antibiotic. This complexation prevented the FRET process by increasing the distance between the CD and MoS_2_, thereby restoring the fluorescence of the CDs (Fig. 13).

**Fig. 14:**
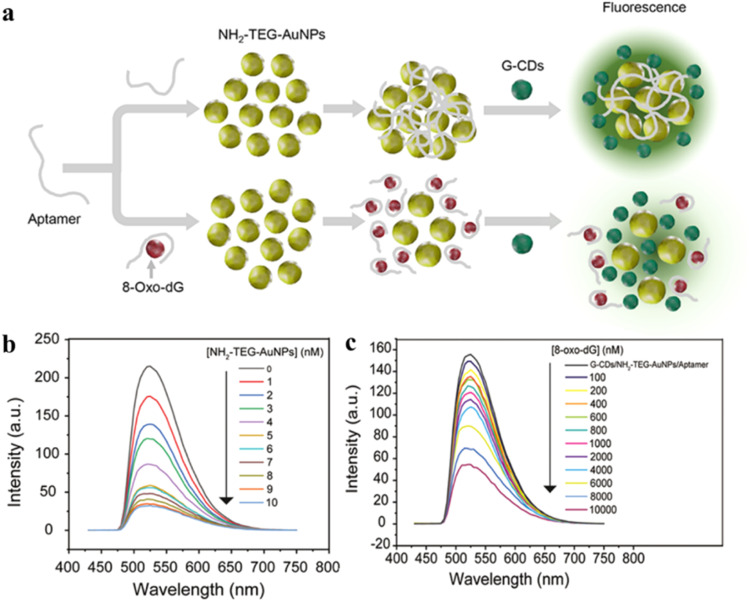
(a) Representative scheme for synthesis and fluorescence detection of 8-oxo-7,8-dihydro-2′-deoxyguanosine. (b) Variation of G-CD fluorescence with varying concentrations of Au NPs, and (c) change of emission signal for the aptamer-conjugated carbon dots upon exposure to 8-oxo-7,8-dihydro-2′-deoxyguanosine. [Reprinted with permission from *ACS Appl. Nano Mater.*, 2023, **6**(8), 7055–7064. Copyright 2023 American Chemical Society].^118^

Composites of carbon dots with gold nanoparticles (Au NPs) have shown great promise in terms of fluorescence sensing. However, most of the synthetic procedures involve addition of separate salt ions for the aggregation of the Au NPs. To overcome this limitation, recently, Ngernpimai and co-workers have synthesized aptamer-loaded amino-terminated oligo(ethylene glycol)-capped gold nanoparticles (NH_2_-TEG-Au NPs) and added gradually to a green CD solution, obtained pyrolytically from citric acid and urea (Fig. 14).^118^ This was utilized for the specific sensing of one of the harmful oxidative degradation products of DNA, *viz.* 8-oxo-7,8-dihydro-2′-deoxyguanosine. In the absence of the analyte, the inherent fluorescence of the green CDs was quenched owing to the close proximity with NH_2_-TEG-Au NPs *via* inner filter effect. When the mixture was exposed to 8-oxo-7,8-dihydro-2′-deoxyguanosine, NH_2_-TEG-Au NPs underwent specific electrostatic interactions with the deoxyguanosine-biomarker, leading to detachment from CDs and aggregation of the NH_2_-TEG-Au NPs and regeneration of the inherent green fluorescence of the CDs. The specific interaction between the aptamer and the deoxyguanosine biomarker showed an excellent detection limit as low as 15.89 nM.

Aptamers and antibodies are the two main types of biomolecules used for biosensing purposes owing to their target-specificity and selectivity. However, aptamers show better stability than antibodies as they can retain their structures against varying temperature, in the presence of chelating ligands, and high salt concentrations. Aptamer production does not involve animal sources, thereby reducing the cost of aptamer-decorated CDs. Hence, aptasensors derived from CDs are superior choices compared to antibody-decorated CDs. Besides aptamers and antibodies, oligonucleotides have been utilized to prepare CDs for biosensing.^119^ They are often introduced on CD surface *via* electrostatic interactions between the negatively charged phosphate group of oligonucleotides and the positive surface charge on CDs. Further exploration can be focused on developing CDs conjugated with other significant biomolecules to ensure selective biosensing.

## Application of carbon dots in the fluorescence detection of diabetes

Diabetes type I and II are caused by two different reasons: type I is caused when pancreas does not produce sufficient insulin owing to the body's autoimmune system attacking islet cells, and type II is caused when the body develops resistance against insulin due to some metabolic disorder. Such unavailability of insulin results in the accumulation of glucose in the bloodstream instead of being distributed to other cells of the body, thereby elevating the glucose level in blood. Therefore, blood-glucose level is one of the most prominent indicators of diabetes. Under physiological conditions, glucose is oxidized to gluconic acid and hydrogen peroxide in the presence of glucose oxidase. Hence, the hydrogen peroxide content in blood is also a significant biomarker for diabetes. Apart from these two, there are several other secondary biomarkers associated with diabetic patients, *e.g.*, enhanced cellular viscosity, elevated hypochlorite level in blood, presence of reactive oxygen species (ROS), *etc.* In the following section, methods of fluorescence-based detection of these biomarkers have been discussed for timely diagnosis and treatment for diabetes.

### Detection of glucose

Selective detection and quantification of glucose is one of the major techniques for detecting diabetes in any patient. Fluorescence-based detection of glucose can be achieved *via* three mechanisms: (i) interaction of glucose with glucose oxidase (GO_*x*_), (ii) quantifying the hydrogen peroxide formed *via* the oxidation of glucose by glucose oxidase, and (iii) interaction of glucose with phenylboronic-acid-type functional groups.

**Fig. 15:**
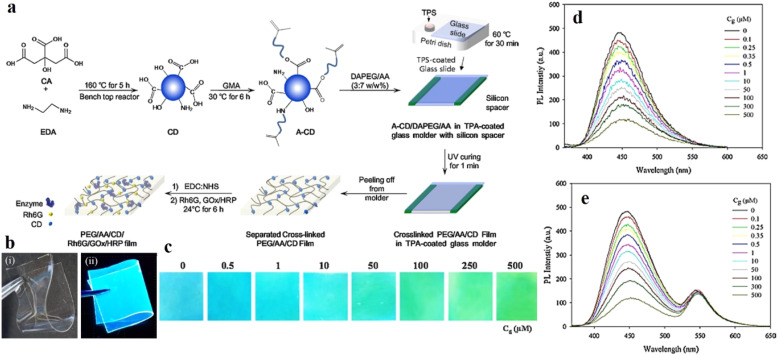
(a) Synthetic scheme of DAPEG/AA/CD/Rh6G/GO_*x*_/HRP film. (b) Photograph of DAPEG/AA/A-CD/Rh6G/GO_*x*_/HRP film under (i) daylight and (ii) UV lamp. (c) Change in fluorescence of the solid film upon interaction with glucose. Fluorescence spectral changes on gradual addition of glucose into (d) CD/GO_*x*_/HR and (e) CD/Rh6G/GO_*x*_/HRP. [Reprinted from *Sensors and Actuators B: Chemical*, **282**, Moon-Jin Cho, Soo-Young Park, Carbon-dot-based ratiometric fluorescence glucose biosensor, 719–729, copyright (2019), with permission from Elsevier].^120^

In this context, Cho and co-workers developed CDs with better longevity as aqueous dispersion using ethylene diamine and citric acid, and as solid films by polymerizing the mixture with glycidyl methyl methacrylate.^120^ The CDs were functionalized with a fluorescent dye rhodamine for constructing a ratiometric probe for better visualization. The CD/rhodamine composite exhibited two distinct emission peaks at around 440 nm and 550 nm upon excitation at 360 nm, corresponding to CDs and rhodamine, respectively. The glucose oxidase (GO_*x*_) and horseradish peroxidase (HRP) composites of the CDs were then synthesized to introduce specific glucose reaction site. For the CD/rhodamine/GO_*x*_ composite, both the emission peaks remain unaffected upon glucose addition; however, for the hybrid composite of CD/rhodamine/GO_*x*_/HRP, there was a sharp drop in CD emission at 440 nm, with the emission at 550 nm remaining unaffected. The ratio of these two emission peaks helped in the selective detection of glucose (Fig. 15).^120^ This observation could be attributed to the interaction of the CDs with hydroxyl radicals, formed from the oxidation of glucose in a hybrid enzymatic medium, with the CDs. Cho observed similar changes in emission property using the CD/rhodamine/GO_*x*_/HRP solid film.

In a recent study, a novel CD-silver nanocomposite (NS-CDs-Ag NPs) was synthesized *via* one-pot microwave irradiation using glutathione and citric acid as the precursors for N, S co-doped carbon dots (NS-CDs) and AgNO_3_.^121^ Doping with Ag initially quenched the fluorescence of NS-CDs. However, owing to the excellent peroxidase mimicking nature, the fluorescence of NS-CDs-Ag NPs was selectively retrieved upon introduction of H_2_O_2_. The fluorescence of NS-CDs-Ag NPs was regenerated *via* the oxidation of Ag by H_2_O_2_ to produce Ag^+^. In addition, the hydroxyl radical (^.^OH) formed from the reaction between NS-CDs-Ag NPs and H_2_O_2_ could promptly oxidize TMB, reflected by a distinct change in its absorption spectrum. This enabled the application of the nanocomposites in colorimetric detection of glucose. These reactions of the NS-CDs-Ag NPs could be extended to the reaction with H_2_O_2_ produced from the interaction between glucose and GO_*x*_. Therefore, NS-CDs-Ag NPs could act as a dual-mode sensor (involving fluorimetric and colorimetric modes) for glucose. The nanocomposites were also utilized for the estimation of blood glucose level with real life blood samples.

**Fig. 16:**
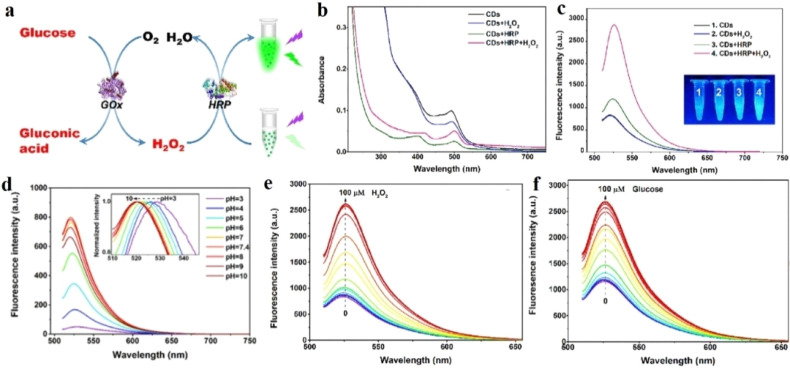
(a) Mechanistic details of fluorescence sensing of hydrogen peroxide and glucose (b) absorbance and (c) emission of the carbon dots in presence of H_2_O_2_, HRP, and both H_2_O_2_ and HRP. (d) pH-dependent emission property of carbon dots. Change in fluorescence response upon successive addition of (e) H_2_O_2_ and (f) glucose. [Reprinted from Yuxuan Hu, Jialin Wen, Dan Li, Yuting Li, Muidh Alheshibri, Minmin Zhang, Lingling Shui, Na Li, Carbon dots-based fluorescence enhanced probe for the determination of glucose, *Spectrochimica Acta Part A: Molecular and Biomolecular Spectroscopy*, **303**, 123149, copyright (2023), with permission from Elsevier].^122^

In a similar line of approach, novel green fluorescent CDs were synthesized by a facile one-pot hydrothermal method using polyethyleneimine and a fluorescent dye, Eosin Y, as the precursors (Fig. 16).^122^ These CDs showed absorption maxima at ∼490 nm with a strong emission peak at 520 nm. Upon gradual addition of H_2_O_2_ and HRP, a prominent red shift was observed in the absorption spectrum, attributed to the enhancement of conjugation in the CDs. Further, TEM images depicted an increase in the size of the nanomaterial in the presence of H_2_O_2_ and HRP, indicating probable aggregation. This observation corroborated with the reduced fluorescence lifetime of the CDs in the presence of H_2_O_2_ and HRP, signifying enhanced radiative transitions. The fluorescence enhancement could also be attributed to the oxidation of the surface functionalities on CDs by reactive oxidative species generated upon enzymatic oxidation of glucose. Hence, this study demonstrated a simple and effective method for determination of biological metabolites related to H_2_O_2_ generation, which exhibited potential for application in clinical diagnosis and research.

In a related study, CDs were introduced on the surface of manganese dioxide (MnO_2_) nanosheets *via* electrostatic interactions.^123^ Owing to the FRET from CDs to the MnO_2_ nanosheets, the emission from CDs gets largely quenched. Upon addition of a mixture of glucose and GO_*x*_, the nanosheets get reduced to Mn^2+^, thus recovering fluorescence. This can be attributed to the interaction of H_2_O_2_, obtained from the reaction between glucose and GO_*x*_, with the nanosheets.

Besides such enzymatic reactions, glucose detection can be achieved in non-enzymatic modes, such as utilization of the reaction of glucose with boronic acids that are susceptible to reaction with diols. Keeping this in mind, there have been attempts to synthesize various phenyl-boronic-acid-functionalized CDs.^124,125^

In one study by Shen *et al.*,^124^ simple CDs were prepared, surface functionalized with boronic acid units.^5^ In this case, phenylboronic acid was directly used as the carbon source for the hydrothermal synthesis of CDs from a solution of NaOH. Upon gradual addition of glucose, the CDs aggregated *via* the formation of boronate complex with the cis diol, *i.e.*, glucose. Fluorescence emissions for the boronic-acid-functionalized CDs was distinctly quenched *via* the mechanism of quenching surface states. This mechanism involves the formation of covalent bond formation between multiple CD units such as glucose containing two pairs of diol moieties.

**Fig. 17:**
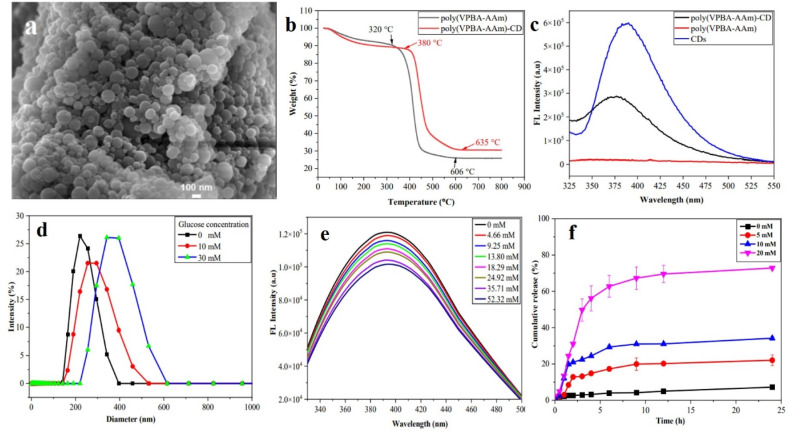
(a) Scanning electron microscopy (SEM) image of the hydrogel-carbon dot (hydrogel-CD) composite. (b) Analysis of thermal stability of the hydrogel-CD composite. (c) Emission properties of hydrogel, CD, and hydrogel-CD composite. (d) Variation in size of the hydrogel on addition of glucose, (e) change in fluorescence spectra of the CD-hydrogel on sequential addition of glucose, (f) release of insulin from the hydrogel-CD composite in the presence of glucose at different concentrations. [Reprinted from J. Zhu, W. Liu, B. Zhang, D. Zhou, X. Fan, X. Wang, X. Liu, Carbon dots embedded hybrid microgel with phenylboronic acid as monomer for fluorescent glucose sensing and glucose-triggered insulin release at physiological pH, *Nanomaterials*, **12**, 3065, copyright (2022), with permission from MDPI].^125^

Zhu and colleagues developed a CD-loaded hydrogel for the detection of glucose and simultaneous delivery of insulin (Fig. 17).^125^ The hydrogel-CD was prepared by radical polymerization between monomers 4-vinylphenylboronic acid (VPBA) and acrylamide (AAm). As glucose concentration exceeded 20 mM, the hydrogel swelled up, along with a gradual decay in fluorescence intensity. This could be attributed to the reaction between glucose and the VPBA units, leading to a change in the Rayleigh scattering intensity of the CD unit or the number of defects on the surface of CDs. The latter produced changes in spin state redistribution and partial charge density, which in turn affected the fluorescence property. These hydrogel-CD composites were also able to form hydrogen bonds with insulin, thereby achieving a loading capacity of 15.1 ± 0.63%. Swelling of the hydrogel-CD hybrid composite with increasing glucose concentration also facilitated the release of insulin.

Several other classes of CDs like polymer CDs, graphene quantum dots, *etc.* have also been utilized for the selective sensing of glucose.^126–128^ Although the enzymatic mode of glucose detection is more specific, with very low limit of detection (LOD), the non-enzymatic mode is cost-effective and more direct. Most of the non-enzymatic detections of glucose involve phenylboronic acid units. Functioning of phenylboronic acids has a crucial dependence on the pH of the medium. Slightly alkaline pH, which favours the formation of the negatively changed boronate anion structure, is the most suitable form of boronic acid for interaction with *cis*-diols. However, at higher alkaline pH, boronic acid may get deprotonated. Thereby, pH optimization is very pivotal to design selective fluorescence sensors for glucose with phenylboronic acid derivatives. Further, the phenylbornic acids have been found to interact with various other biologically relevant *cis*-diols, which limits their application. Hence, there is an urgent need for the development of new more selective and sensitive non-enzymatic pathways to improve the scope of the CD-based probes. Functionalities, other than phenylboronic acid groups, which can react with glucose selectively, can be examined for the development of better CD-based fluorescent probes.

### Detection of hydrogen peroxide

Hydrogen peroxide (H_2_O_2_) is a significant biomarker for diabetes. For patients with type II diabetes, large amounts of reactive oxygen species (ROS) are generated with a slow rate of physiological response, which ultimately results in very high oxidative stress. H_2_O_2_, being one such ROS, is found in large excess in the bloodstream of diabetic patients. Such excess H_2_O_2_ not only causes high oxidative stress but is also responsible for malfunctioning or destruction of beta cells in pancreas, thus restricting the formation of insulin.^129,130^ It is therefore imperative to selectively detect and quantify intracellular H_2_O_2_ under physiological conditions.

**Fig. 18:**
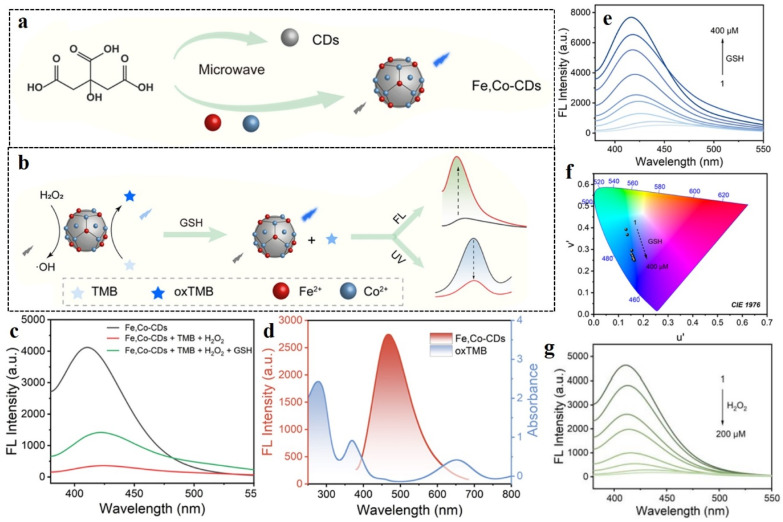
(a) Microwave-mediated production of Fe and Co-doped carbon dots. (b) Schematic representation of the peroxidase activity in terms of response to H_2_O_2_ and GSH, (c) fluorescence property of Fe, Co-doped carbon dots in the presence of TMB, H_2_O_2_, and GSH, (d) overlap between the absorption spectrum of TMB with the emission spectrum of the carbon dots, (e) increase in fluorescence intensity for the Fe, Co-doped carbon dots on gradual addition of GSH, (f) CIE chromaticity diagram for implying the change in color for Fe, Co-doped carbon dots, (g) decrease in fluorescence intensity for Fe, Co-doped carbon dots on successive increase in the concentration of H_2_O_2_. [Reprinted with permission from *ACS Appl. Nano Mater.*, 2024, **7**(15), 17795–17803. Copyright 2024 American Chemical Society].^131^

A recent study by Dai *et al.* elaborated the application of metal-doped CDs for the simultaneous determination of intracellular H_2_O_2_ and glutathione content.^131^ Dai *et al.* prepared bifunctional CDs doped with Fe and Co. The doped CDs showed blue fluorescence, which was quenched in the presence of 3,3′,5,5′-tetramethylbenzidine (TMB) owing to FRET from the CDs to the TMB unit (Fig. 18). Owing to their ability to catalytically degrade H_2_O_2_ to hydroxyl radicals, the doped CDs could serve as peroxidase mimicking agents. Upon addition of H_2_O_2_, the fluorescence of the CDs could be restored owing to the oxidation of TMB by hydroxyl radical. On further addition of glutathione, the fluorescence of the CDs experienced a sharp decline with enhancement in absorption due to the termination of the catalytic oxidation of TMB. The limit of detection for H_2_O_2_ was calculated to be 0.81 µM, which was well within the range of intracellular H_2_O_2_ concentration.

A similar approach was followed by Ngo *et al.*; they developed nitrogen-doped CDs for the sensing of H_2_O_2_ from uric acid and glucose precursors *via* imine bond formation.^132^ However, to further improve the optical and catalytic properties of the CDs, the nitrogen-doped CDs were functionalized with metal oxides like manganese oxide and ferric oxide (MNFCDs). The sensing of H_2_O_2_ was monitored using TMB as in the previous case. Ngo *et al.* explained that the diamine form of TMB can be oxidized to form two intermediate oxidation products: (i) a colorless TMB radical cation, produced upon one electron transfer, and (ii) a charge transfer complex (CTC) (intermediate oxidation state), produced upon two-electron transfer. The CTC showed absorption maximum at 652 nm. After the completion of two-electron oxidation, the blue CTC was converted to a yellow oxidation product, which absorbed at 450 nm. Based on these distinct color changes, the presence of H_2_O_2_ was detected with precision. Many other researchers have reported such selective detection of H_2_O_2_ in terms of the peroxidase mimicking property of CDs *via* the oxidation of TMB.^133,134^

**Fig. 19:**
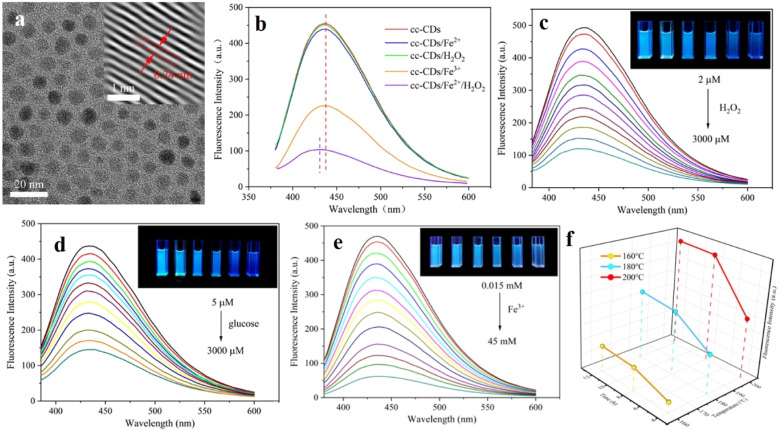
(a) Characterization of carbon dots obtained from chicken cartilage by TEM image. (b) Change in emission properties of the carbon dots in presence of Fe^2+^, H_2_O_2_, and Fe^3+^. Gradual decrease in the fluorescence intensity with increasing amounts of (c) H_2_O_2_, (d) glucose, and (e) Fe^2+^. (f) Temperature- and time-dependent increment of fluorescence for the carbon dots. [Reprinted from Lei Wu, Wenyue Pan, Heng Ye, Ning Liang, Longshan Zhao, Sensitive fluorescence detection for hydrogen peroxide and glucose using biomass carbon dots: dual-quenching mechanism insight, *Colloids and Surfaces A: Physicochemical and Engineering Aspects*, **638**, 128330, copyright (2022), with permission from Elsevier].^135^

Following a greener approach, Wu *et al.* prepared a new class of CDs from chicken cartilage.^135^ Presence of abundant heteroatoms in chicken cartilage in the biomass precursor, renders the CDs with intriguing optical and catalytic properties. These hydrothermally synthesized green CDs possessed hydrophilic functional groups like OH, NH, *etc.* on their surface, making them highly water-soluble and biocompatible. In this case, Fe^2+^ initiates the H_2_O_2_ sensing process. A mixture of H_2_O_2_ and Fe^2+^, often known as Fenton's reagent, generates the hydroxyl radical, which in turn quenches the fluorescence of the CDs (Fig. 19). With increasing concentration of both Fe^2+^ and H_2_O_2_, the fluorescence emission of CDs was found to decrease to a great extent, with a very low detection limit of 0.47 µM.

Although CDs are being extensively employed for fluorescence-based detection of H_2_O_2_, they often suffer from lack of selectivity. All the existing mechanisms involved in H_2_O_2_ detection mostly rely on the oxidation of the surface functionalities on the CDs. However, other reactive oxygen species present in the physiological system of a diabetes patient may also bring about such oxidation. Therefore, the selectivity towards H_2_O_2_ is reduced. This lack of selectivity can be addressed by decorating the CDs with specific functionalization like antibodies, enzymes, *etc.* Moreover, reactions of CDs with H_2_O_2_ may lead to various other side reactions, leading to complicated and uncontrollable optical changes. Often, the H_2_O_2_ detection modes involve Fenton type reactions for which CDs are doped with selective metal ions like Fe^2+^. Incorporation of metal ions may impact the biocompatibility of the CDs. Further, application of CD-based nanomaterials for the fluorescence-based detection of H_2_O_2_ is restricted only up to certain nanomolar levels. The physiological microenvironment consists of H_2_O_2_ at levels as low as the picomolar scale, which may not be efficiently detected by the existing CD materials. Hybridizing CDs with other reactive nanomaterials like metal organic frameworks can be a promising alternative in this respect. Despite these limitations, CDs are being widely explored for fluorescence detection of H_2_O_2_ due to their scalable, low-cost preparations, and excitation-independent fluorescence.

### Detection of hypochlorous acid (HClO)

Hypochlorous acid (HClO) is one of the ROSs generated in the endoplasmic reticulum, lysosome, or mitochondria of diabetic patients, from the reaction between hydrogen peroxide and chloride ions, catalyzed by the heme peroxidase enzyme, *i.e.*, myeloperoxidase. When present in the appropriate concentration (<200 µM),^136^ HClO helps prevent the invasion of microorganisms; however, a high concentration of HClO results in cell apoptosis. Therefore, facile detection of HClO is of high significance in the diagnosis of diabetes.

**Fig. 20:**
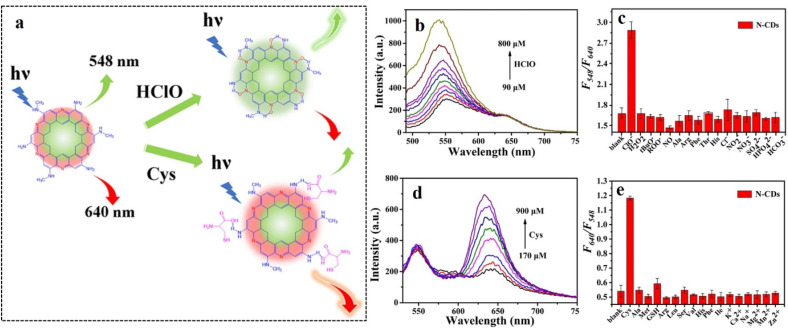
(a) Demonstration of fluorescence sensing of hypochlorous acid and glutathione by nitrogen-doped carbon dots. (b) Sharp increase in emission intensity of the nitrogen-doped carbon dots with successive addition of hypochlorous acid, (c) comparative study of the change in emission intensity in the presence of other interfering reactive anions, (d) sharp increase in emission intensity of the nitrogen-doped carbon dots with successive addition of glutathione, (e) comparative study of the change in emission intensity in the presence of other amino acids. [Reprinted from Z. Wu, M. Cai, W. Lv, C. Lu, B. Wu, C. Ren, Y. Dong, H. Chen, X. Chen, A fluorescence “turn-on” probe for the respective ratiometric detection of hypochlorite and cysteine, *Sensors & Diagnostics*, **2**, 1207–1214, copyright (2023), with permission from Royal Society of Chemistry].^137^

In a recent study, Wu *et al.* prepared nitrogen-doped CDs and utilized the dual emission of the nitrogen-doped CDs at 548 nm and 640 nm to curate a sensitive ratiometric probe for the simultaneous detection of HClO and cysteine.^137^ The synthesis of the CDs was achieved using *N*-methylbenzene-1,2-diamine as the precursor after a thorough optimization process. The emission peak at 548 nm underwent blue shift with enhanced intensity upon interaction with HClO (Fig. 20). A similar blue shift from 554 nm to 520 nm was observed in the absorption spectrum. Solvatochromic study in the presence of a series of polar protic (dimethyl formamide and dimethyl sulfoxide) and aprotic (ethanol, methanol, *etc.*) solvents revealed that the absorbance of CDs was influenced by the solvent polarity. Hence, the fluorescence of these nitrogen-doped CDs was attributed to their molecular state, *i.e.*, the fluorophore functionalities at the core of the CDs. The blue shifts in both the absorption and emission spectra were indicative of an internal charge transfer (ICT) process. The fluorescence switching mechanism, caused by the restriction on the ICT process, was further supported by theoretical calculations of the HOMO–LUMO energy levels.

Another prototype fluorescent probe for HClO was reported by Zou *et al.* They synthesized novel iron-oxide-doped magnetic CDs, which showed excellent site-specificity towards lysosome.^138^ The CDs were initially synthesized by the hydrothermal reduction of 1,3,6-trinitropyrene (TNP) in the presence of sodium borohydride; these CDs were then doped with magnetic Fe_3_O_4_*via* electrostatic self-assembly. Addition of ClO^−^ resulted in two new peaks at around 490 and 549 nm, owing to improved conjugation in the CD structure. Additionally, the magnetic CDs underwent a quenching in fluorescence intensity, which was attributed to the oxidation of the amine groups to nitro groups on the surface of the CDs. This was further confirmed by the appearance of new peaks at 1345 cm^−1^ and 645 cm^−1^ in the IR spectrum, corresponding to N–O and C–Cl stretching modes, respectively. The LOD for these lysosome-targeted CDs was found to be as low as 0.27 µΜ. Lysosomes being one of the most important sites for the synthesis of ClO^−^, close monitoring of the level of ClO^−^ in lysosomes is of utmost importance. In this regard, the reported CDs proved very useful for the fluorescence detection of ClO^−^ for the timely diagnosis of diabetes and other health hazards.

**Fig. 21:**
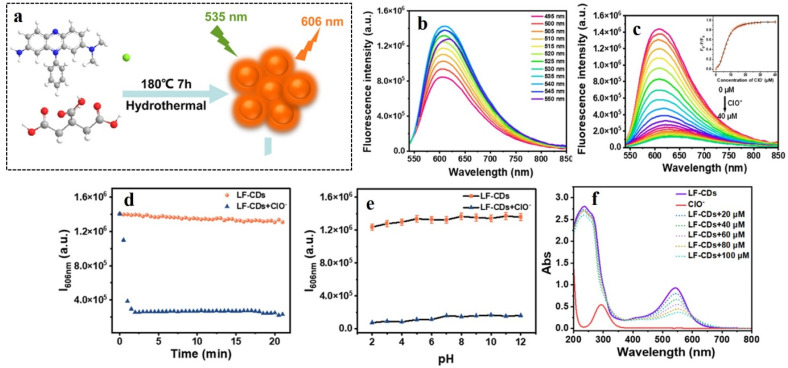
(a) Hydrothermal synthesis of long-wavelength fluorescence-emission carbon dots (LF-CDs) from citric acid and basic violet 8. (b) Excitation-wavelength-dependent emission of carbon dots, (c) quenching of fluorescence of carbon dots with increasing concentration of hypochlorite, (d) time-dependent and (e) pH-dependent variation of emission intensity for LF-CDs at 660 nm, (f) comparative absorption spectra of LF-CDs, hypochlorite ion, and both LF-CDs and ClO^−^. [Reprinted from Xia Bian, Xia Lu, Yongfei Huang, Xiaoye Wen, Zhefeng Fan, Development of non-modified long-wavelength fluorescent-emission carbon dots for the imaging of intracellular hypochlorous acid (ClO^−^) during ferroptosis, *Sensors and Actuators B: Chemical*, **428**, 137265, copyright (2025), with permission from Elsevier].^139^

The iron-dependent apoptosis method, known as ferroptosis, affects the pathogenesis of diabetes, leading to diabetic retinopathy or nephropathy. Hence, a detailed study on the ferroptosis mechanism can prove helpful for diabetes treatment. Therefore, considerable research effort has recently been directed to the detection of different diabetic biomarkers during the ferroptosis process. In this attempt, to design a new class of CDs with longer emission wavelengths, Bian *et al.*^139^ synthesized CDs from a prominent fluorescent dye basic violet 8 and citric acid by a one-step hydrothermal technique. The CDs showed two prominent absorption peaks at ∼255 and ∼542 nm, corresponding to π–π* and *n*–π* transitions, respectively. With increasing amount of ClO^−^, there was a decrease in the intensity of the peak at 542 nm, indicating loss of lone pairs of electrons from the surface of the CDs. Further, no overlap between the absorption spectrum of ClO^−^ with either the excitation or the emission spectra of the CDs suggested that there were no energy transfer processes associated with inner filter effect. Further, insignificant changes in the fluorescence lifetime indicated the involvement of a static quenching mode. Thus, the decrease in fluorescence intensity of the CDs were attributed to the oxidation of the amine groups on the surface of CDs to nitro group upon interaction with ClO^−^ (Fig. 21).

From the aforementioned discussion, it is evident that CDs have considerable potential for application in the selective detection of HClO/ClO^−^ ratio. Although there has been extensive research aimed at developing fluorescent probes for HClO or ClO^−^, utilizing CDs for the same is still not sufficiently explored. Possible reasons behind this can be the difficulty in isolating and purifying CDs, lack of clear understanding of the detection mechanism, *etc.* In most of the reported studies CDs undergo a steep fluorescence quenching on interaction with HOCl, which leads to inefficient visualization, sensitive to other environment-dependent variations. In addition, optical response from CDs on reaction with HOCl often stems from oxidative degradation or permanent conversion of surface states of CDs, which restricts the reversibility of the mechanism. To address these issues, a wide range of functionalities are being explored to improve the sensitivity and selectivity of CD materials. One of the promising approaches can be to design CD-based probes, which are able to show fluorescence lifetime-based response bypassing the intensity-dependence. Further development in this area would greatly help the early diagnosis of diabetes.

### Other biomarkers

Besides the prominent biomarkers mentioned above, several other biomarkers are available, which also help indicate diabetes. Researchers have started exploring the detailed metabolic reactions in diabetic patients and have successfully identified new biomarkers. Peroxynitrite is one such example, which belonged to the class of ROSs. Other than that alkaline phosphate, certain ketones, intracellular viscosity, *etc.* influence the microenvironment in diabetic patients.

Ketones like acetone are produced in the blood stream during a condition called diabetes ketoacidosis, in which the liver produces ketone as an alternate source of energy as the glucose level drops in cells. Fluorescent probes capable of detecting acetone can be utilized to design breath analyzers for the early detection of diabetes. In one of their recent findings, Alshareef *et al.* fabricated nitrogen-doped CD-based testing strips for the detection of acetone with LOD as low as 1.8 ppm.^140^ The nitrogen-doped CDs were synthesized by a hydrothermal method from cellulose diacetate prepared from waste rice straw. The CDs were then cast on Whatman filter paper by dipping the filter paper into a series of solutions of CDs in the presence of phosphate buffer. Upon gradual increase in the acetone content, the CD-caste paper strips showed a decay in fluorescence intensity attributed to FRET from the nitrogen-doped CDs to the acetone upon physisorption of acetone on the paper strips. These paper strips were found to produce highly reversible and durable results in the presence or absence of acetone.

**Fig. 22:**
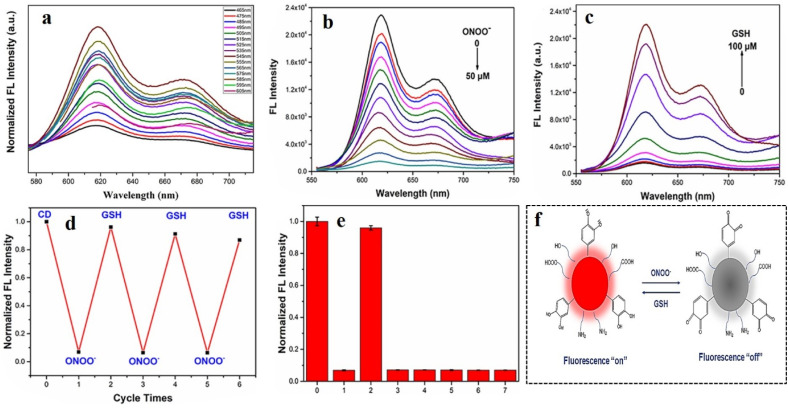
(a) Excitation-wavelength-dependent fluorescence spectra for nitrogen and zinc-doped carbon dots. (b) Quenching of fluorescence for the carbon dots upon successive addition of peroxynitrite, (c) gradual increase in the fluorescence intensity with increasing glutathione concentration, (d) recovery cycle of the fluorescence of carbon dots with periodic addition of peroxynitrite and glutathione, (e) fluorescence recovery study in the presence of different antioxidants like (1) only carbon dots, (2) glutathione, (3) cysteine, (4) homocysteine, (5) H_2_S, (6) ascorbic acid, (7) glucose. (f) Representation of the reversible change in fluorescence property of nitrogen, zinc-doped carbon dots. [Reprinted from Meng-Jie Fu, Na Wei, Lan-Fang Pang, Xiao-Feng Guo, Hong Wang, Red emission nitrogen and zinc co-doped carbon dots as fluorescent sensor for reversible detection of peroxynitrite in living cells, *Sensors and Actuators B: Chemical*, **351**, 130939, copyright (2022), with permission from Elsevier].^141^

Reactive nitrogen species (RNS) are another class of biomarkers found in excess in diabetes patients. In the search for fluorescent probes for detecting diabetes-related RNS like peroxynitrite/glutathione, Fu *et al.* developed nitrogen- and zinc-doped CDs for reversible fluorescence detection of peroxynitrite ions (Fig. 22).^141^ The CDs were synthesized by hydrothermal reaction between catechol and 3,4-diaminobenzoic acid to achieve maximum conjugation. Greater conjugation afforded CDs showing red fluorescence, which is free from any interference. The catechol groups on the surface of the CDs, capable of quinine–phenol interconversion under suitable redox environment, resulted in reversible changes in fluorescence. The fluorescence properties of these CDs were further improved by the introduction of zinc on the surface. Upon interaction with peroxynitrite, the fluorescence of the CDs was reduced with an LOD of 0.6 µM. This quenching of fluorescence could be attributed to the oxidation of catechol group to quinine structure, as also supported by the disappearance of the peak at 3396 cm^−1^ in the IR spectrum. However, further interaction with glutathione restored the fluorescence *via* quinine–phenol transformation.

The most frequently used diabetes monitoring systems till date include wide application of glucose testing strips. Although handy and portable, these strips are expensive and invasive. These strips constitute specific enzymes grafted on electrodes, which may cause restricted reproducibility. Nonetheless, these strips can trace ultra-low levels of glucose with long-term stability. CD-based optical sensors, on the other hand, entail biocompatible and non-invasive detection. CD-based optical sensors not only facilitate real-time monitoring of glucose but can also specifically detect other crucial biomarkers related to diabetes like oxidative stress, hydrogen peroxide, *etc.*, which in turn help in identifying diabetes at its inception. Other FDA-approved commercial diabetes detection methods encompass HbA1c assay, continuous glucose monitoring, *etc.* Although CGM involves continuous real-time monitoring of glucose, this method requires regular replacement owing to the degradation of constituent enzymes. Both CD-based optical sensors and CGM can quantify glucose up to as low as nanomolar level. Even though HbA1c assay can accurately monitor long-term change in glucose level, it is not suitable for daily glucose monitoring.

Keeping these in mind, most of the recent research delves into widespread application of CDs for the fluorescence-based detection of diverse range of diabetes biomarkers. However, inherent toxicities of some of the CDs restrict their application in biosensing. Hence, exploration of greener methods for the synthesis of CDs with minimum post-synthetic treatments may facilitate their application in biosensing. Another shortcoming stems from the long-term retention of CDs in physiological medium, which leads to undesired side effects. Further research must be focused on the removal of CDs from the physiological system for better *in vivo* applications leading towards clinical approval. Additionally, over-reliance on the reaction conditions limits scalability and commercialization of CD-based optical sensors. Slight change in temperature or reaction time may affect the size, shape, or morphology of CDs to a great extent. Although there have been a few attempts to develop CD-based paper strips for glucose monitoring, further studies are required before successful commercial translation. Table 2 presents a compartive analysis of CD-based glucose sensors and commercially available enzyme-based sensors.

**Table 2:** Comparative study of state-of-art CD-based glucose sensors with commercially available enzyme-based sensors

Type of sensor	Method of sensing	Limit of detection (LOD)	Linear range	Selectivity	Response/recovery time	Stability	Advantage	Disadvantage	Clinical applicability	Refs
CD/Rh6G/GO_*x*_/HRP	Ratiometric fluorescence	0.04 µM	0.1–500 µM	Good selectivity against Na^+^, K^+^, cholesterol, ascorbic acid *etc.*	Solution: ∼102%; film: ∼105%	Better long-term storage of the solid-state hydrogel film	Very low LOD, dual response of colorimetric and fluorescence	Narrow linear range	Potential candidate for *in vitro* serum assays	120
N,S-codoped CD/Ag composite	Fluorimetric and colorimetric	>0.21 mM	Fluorimetric mode: 0.1–80 µM, colorimetric mode: 0.5–5 µM	Highly selective even in presence different metal ions and amino acids	Fluorimetric mode: 91–100%, colorimetric mode: 95–99%	Resistant to acid, alkali, salt, temperature effects	High recovery with human serum sample	Undesired redox reactions	Good peroxide activity	121
N,S-codoped CD-microgel	Fluorescence	20 mM	0–29.28 mM	Influenced by metal ions like Fe^3+^, Hg^2+^	—	Thermally stable up to 380 °C	Easy one-pot synthesis	pH-dependent swelling–deswelling	Glucose concentration-dependent insulin release	125
CD/CuO composite	Electrochemical	1.4 nM	∼15–225 nM	High selectivity towards glucose in alkaline conditions at a fixed potential of 0.5 V	99–102%	High electrode stability maintaining 99% of current over prolonged periods	Cost-effective, high stability, wider operational temperature range	Synthesis involves high temperature heating	Real blood serum detection	142
Carbon quantum dot	Fluorescence	165 µM	0.165–8 mM	Good selectivity against Na^+^, K^+^, amino acid, urea, other saccharides *etc.*	98–107%	Photostability up to two months	Resistant to photobleaching	Use of large excess of organic solvent for the pulsed laser fragmentation synthesis	Real human saliva analysis	143
GO_*x*_/graphitic carbon nitride quantum dots (CNQDs)/PANI	Enzymatic electrochemical biosensing	0.029 µM	0–0.5 mM	Highly selective glucose oxidation in presence of uric acid, ascorbic acid, and urea	96.27%	High selectivity and repeatability	Miniaturization, repeatability, selectivity, fast response time	Sensitive to pH and temperature	Wearable sweat biosensor	144
Cu@NCQD	Fluorescence	29.85 mM	0–140 mM	High selectivity in presence of ascorbic acid, dopamine, fructose	—	Profound stability, repeatability, and deposition time	Increased selectivity towards glucose due to strong binding of Cu^2+^ ions to glucose and facile electron transport on carbon dot surfaces	Influence of polarity of the medium owing to the presence of highly polar surface functional groups	Ease of application and cost-effective	145
Contour plus blood glucose test strips	Enzymatic electrochemical biosensing	∼0.2–0.6 mM	1.1–33.3 mM	—	Measuring time: 5 s, second chance sampling	18–24 months	Small sample size within 0.6 mL, automatic haematocrit correction	Restricted to high (>5 mM) glucose, invasive	Meets ISO standards with a high percentage of results within ±15 mg dL^−1^ of lab reference	146
Dr Morepen gluco one glucose strips BG-03	Enzymatic electrochemical biosensing	∼0.2–0.6 mM	1.1–33.3 mM	—	Measuring time: 5 s	Single use strip	Small sample size within 0.5 mL	Low accuracy	*In vitro* diagnostic use only	147
Senseonics	Fluorescent, hydrogel-based sensor	20 mg dL^−1^	40–400 mg dL^−1^	Highly selective	Send glucose data to a smartphone app every 5 minutes	Up to 365 days	Longest sensor life, implantable design	Surgical insertion and removal process	Continuous glucose monitoring (CGM) for a prolonged time over one year	148

## Conclusions and future outlook

Increasing rate of insulin resistance, leading to high diabetes levels, has emerged as a matter of serious concern these days. Irregularity in the synthesis and metabolism of insulin usually impact human health adversely, affecting heart, kidney, eyes, bones, *etc.* A timely assessment of the disease and initiating the treatment may greatly reduce the risk factors. Among the diverse detection methods available, fluorescence signaling has been well known in the field of biosensing of several critical diseases owing to the precision and non-invasiveness of the method. A wide range of nanomaterials have been regularly utilized in biomedicinal research owing to their small size, large surface area, porous nature, biocompatibility, *etc.* Carbon-based nanomaterials like CDs have emerged as one of the pioneering materials. The advantages of using CDs lie in their smaller (<10 nm) size, zero-dimensional nature, water-solubility, and fascinating optical properties. Doping of other heteroatoms like nitrogen, sulfur, *etc.* or metal atoms like copper, iron, *etc.* have also been found to improve these properties by introducing new energy levels.

This article discusses different types of CDs, highlighting their synthetic strategies and different electronic and optical properties. Comparative studies revealed that pristine CDs suffer from low quantum yields; however, introduction of a suitable dopant reduced the band gap, affording high quantum yield and excitation-dependent emission properties. CDs have long been in use for biosensing. The biosensing abilities of CDs have been discussed in detail, emphasizing the effects of surface functionalities. Literature review suggested that the biosensing properties of CDs can be modulated either by conjugating with electron-donating and electron-accepting organic molecules or by surface functionalization with important biomolecules like aptamers, antibodies, *etc.* Such typically curated CDs exhibited excellent target-specific emission properties, which is a primary requisite for selective biosensing. Finally, this article reviews some of the recent reports on detecting selective diabetes biomarkers, *e.g.*, glucose, ROSs like hydroxyl radicals, superoxide, singlet oxygen, hydrogen peroxide, *etc.* Detailed mechanistic analysis of the sensing of diabetes biomarkers guided by the change in emission signals of CD conjugates has also been provided in this article.

Despite several advantages, CD-based nanomaterials face numerous constraints in terms of their critical synthetic procedures with low yield and non-uniform shapes and sizes. Scalable green modes of synthesizing CDs needs more attention in view of the development of clinical-grade CDs. Another major drawback for CDs is their extended emission spectra, which restricts the selectivity of their sensing abilities. The tunable optical property of CDs makes them a promising tool for real time monitoring leading to point of care applications. However, most reported CD-based materials absorb either in the UV or visible range; however, they do not show strong absorption in the near-infrared (NIR) region, more specifically in the NIR II region, which is the optimum window for effective biosensing owing to deep tissue penetration and less scattering of the NIR light. Hence, extensive research is required to develop new class of CD materials for NIR II-responsive biosensing. Therefore, this review article will be able to encourage researcher working in the field of nanomaterial-based biosensing to explore advance ways of personalized treatments for diabetes.

## Author contributions

Lina Anil Kumar and Kalyani Priya Manoj: conceptualization and writing original draft (equally contributed); Ananya Kannamvelli Illam and Angel Mariyam Biju: data curation; M. M. Sreejaya: editing and writing original draft; Vaishnavi Mekkeparambath: data curation and writing original draft; Teresa Aditya: supervision and editing; and supervision, review & editing: Moumita Gangopadhyay.

## Conflicts of interest

There are no conflicts to declare.

## Data Availability

No primary research results, software or code have been included and no new data were generated or analysed as part of this review.
